# Effect of Implementation Facilitation to Promote Adoption of Medications for Addiction Treatment in US HIV Clinics: A Randomized Clinical Trial

**DOI:** 10.1001/jamanetworkopen.2022.36904

**Published:** 2022-10-17

**Authors:** E. Jennifer Edelman, Geliang Gan, James Dziura, Denise Esserman, Elizabeth Porter, William C. Becker, Philip A. Chan, Deborah H. Cornman, Christian D. Helfrich, Jesse Reynolds, Jessica E. Yager, Kenneth L. Morford, Srinivas B. Muvvala, David A. Fiellin

**Affiliations:** 1 Program in Addiction Medicine, Yale School of Medicine, New Haven, Connecticut; 2 Department of Internal Medicine, Yale School of Medicine, New Haven, Connecticut; 3 Center for Interdisciplinary Research on AIDS, Yale School of Public Health, New Haven, Connecticut; 4 Yale Center for Analytic Sciences, Yale School of Public Health, New Haven, Connecticut; 5 Department of Emergency Medicine, Yale School of Medicine, New Haven, Connecticut; 6 Department of Biostatistics, Yale School of Public Health, New Haven, Connecticut; 7 VA Connecticut Healthcare System, West Haven; 8 Department of Medicine, Brown University, Providence, Rhode Island; 9 Institute for Collaboration on Health, Intervention, and Policy (InCHIP), University of Connecticut, Storrs; 10 University of Washington and VA Puget Sound, Seattle; 11 SUNY Downstate, Brooklyn, New York; 12 Department of Psychiatry, Yale School of Medicine, New Haven, Connecticut

## Abstract

**Importance:**

Medications for addiction treatment (MAT) are inconsistently offered in HIV clinics.

**Objective:**

To evaluate the impact of implementation facilitation (hereafter referred to as “facilitation”), a multicomponent implementation strategy, on increasing provision of MAT for opioid use disorder (MOUD), alcohol use disorder (MAUD), and tobacco use disorder (MTUD).

**Design, Setting, and Participants:**

Conducted from July 26, 2016, through July 25, 2020, the Working with HIV Clinics to adopt Addiction Treatment using Implementation Facilitation (WHAT-IF?) study used an unblinded, stepped wedge design to sequentially assign each of 4 HIV clinics in the northeastern US to cross over from control (ie, baseline practices) to facilitation (ie, intervention) and then evaluation and maintenance periods every 6 months. Participants were adult patients with opioid, alcohol, or tobacco use disorder. Data analysis was performed from August 2020 to September 2022.

**Interventions:**

Multicomponent facilitation.

**Main Outcomes and Measures:**

Outcomes, assessed using electronic health record data, were provision of MAT among patients with opioid, alcohol, or tobacco use disorder during the evaluation (primary outcome) and maintenance periods compared with the control period.

**Results:**

Among 3647 patients, the mean (SD) age was 49 (12) years, 1814 (50%) were Black, 781 (22%) were Hispanic, and 1407 (39%) were female; 121 (3%) had opioid use disorder, 126 (3%) had alcohol use disorder, and 420 (12%) had tobacco use disorder. Compared with the control period, there was no increase in provision of MOUD with facilitation during the evaluation period (243 patients [27%; 95% CI, 22%-32%] vs 135 patients [28%; 95% CI, 22%-35%]; *P* = .59) or maintenance period (198 patients [29%; 95% CI, 22%-36%]; *P* = .48). The change in provision of MAUD from the control period to the evaluation period was not statistically significant (251 patients [8%; 95% CI, 5%-12%] vs 112 patients [13%; 95% CI, 8%-21%]; *P* = .11); however, the difference increased and became significant during the maintenance period (180 patients [17%; 95% CI, 12%-24%]; *P* = .009). There were significant increases in provision of MTUD with facilitation during both the evaluation (810 patients [33%; 95% CI, 30%-36%] vs 471 patients [40%; 95% CI, 36%-45%]; *P* = .005) and maintenance (643 patients [38%; 95% CI, 34%-41%]; *P* = .047) periods.

**Conclusions and Relevance:**

In this randomized clinical trial, facilitation led to increased provision of MTUD, delayed improvements in MAUD, and no improvements in MOUD in HIV clinics. Enhanced strategies, potentially including clinic and patient incentives, especially for MOUD, may be needed to further increase provision of MAT in HIV clinics.

**Trial Registration:**

ClinicalTrials.gov Identifier: NCT02907944

## Key Points

**Question:**

Does implementation facilitation promote increased adoption of medications for opioid, alcohol, and tobacco use disorder in HIV clinics?

**Findings:**

In this randomized clinical trial of 3647 patients with opioid, alcohol, or tobacco use disorder, during short-term follow-up compared with the control period, implementation facilitation was not associated with a statistically significant increase in observed provision of medication for opioid use disorder (27% vs 28%) or alcohol use disorder (8% vs 13%). There was a significant increase in provision of medication for tobacco use disorder (33% vs 40%).

**Meaning:**

These findings suggest that implementation facilitation can increase provision of medications for alcohol and tobacco use disorder in HIV clinics, although additional efforts may be needed to improve its impact, especially for medications for opioid use disorder.

## Teaser

This randomized clinical trial evaluates the impact of implementation facilitation, a multicomponent implementation strategy, on increasing provision of medications for addiction treatment for opioid, alcohol, and tobacco use disorders.

## Introduction

Substance use disorders (SUDs), including opioid use disorder (OUD), alcohol use disorder (AUD), and tobacco use disorder (TUD), are major factors associated with morbidity and mortality among individuals with HIV. Furthermore, untreated SUDs are associated with risk behaviors and ongoing HIV transmission to threaten public health. Medications for addiction treatment (MAT) for OUD (MOUD), AUD (MAUD), and TUD (MTUD) are safe, effective, and recommended by clinical guidelines for individuals with HIV.^1^ It is recommended that MAT is offered with HIV care to maximize reach to patients and improve clinical outcomes.^1^

Despite the urgent need to intervene to prevent harms associated with SUD, individuals with HIV are infrequently prescribed MAT.^2,3^ This is due, in part, to lack of training and comfort among HIV clinicians.^4,5^ Implementation facilitation (hereafter referred to as “facilitation”), is defined as “a multi-faceted process of enabling and supporting individuals, groups, and organizations in their efforts to adopt and incorporate clinical innovations in routine practices”^6^ and is an effective implementation strategy for improving treatment of chronic conditions in primary care settings.^7,8^ To our knowledge, only a few prior studies have applied facilitation^9^ or any of its components (ie, academic detailing)^10,11^ to promote MAT provision, and there are no published studies in HIV clinics specifically.^12^

Thus, we conducted the Working with HIV clinics to adopt Addiction Treatment using Implementation Facilitation (WHAT-IF?) study to examine the impact of facilitation on promoting MAT provision and increasing clinician, staff, and organizational readiness to promote MAT in 4 diverse HIV clinics in the northeastern US. We hypothesized that facilitation would improve MAT provision among patients with OUD, AUD, or TUD.

## Methods

### Study Design Overview

As described elsewhere,^13,14^ the WHAT-IF? study used a hybrid type 3 effectiveness-implementation design^15^ with a stepped wedge approach^16^ to evaluate the impact of facilitation on promoting provision of MAT and counseling to address OUD, AUD, and TUD in HIV clinics. Study outcomes included provision of MAT (primary) and clinician, staff, and organizational readiness to provide such treatments (secondary). The study was approved by institutional review boards at Yale University and each of the participating universities and health care sites. A waiver of informed consent was obtained because the study involved minimal risk to patients and obtaining consent would have not been practical. The study protocol is shown in Supplement 1. This randomized clinical trial follows the Consolidated Standards of Reporting Trials (CONSORT) reporting guideline for trial studies (eFigure 1 in Supplement 2).^17^

### Study Context and Participants

The study was conducted within the Yale CIRA (Center for Interdisciplinary Research on AIDS) New England HIV Implementation Science Network.^18^ The coordinating center is located at Yale School of Medicine in New Haven, Connecticut, and the Yale Center for Analytic Science coordinated the data management and analytic support. Study activities occurred at 4 urban HIV clinics intentionally selected given their variability in terms of affiliations (ie, academic vs community-based hospital clinic), infrastructure (eg, on-site behavioral health programs), and resources (eg, external grant funding).

### Patient Participants

We extracted electronic health record (EHR) data on all patients with HIV receiving care in the participating clinics from July 26, 2016, through July 25, 2020. Patients were considered to be receiving care if they had a scheduled visit at the clinic during the time period of interest, regardless of attendance, and they were eligible to enter the cohort (ie, open cohort design) at any point during the study period upon meeting these inclusion criteria. Patients were considered to have OUD, AUD, or TUD according to documentation on the problem list, encounter reason, or international diagnostic codes (*International Classification of Diseases, Ninth Revision* and *International Statistical Classification of Diseases and Related Health Problems, Tenth Revision*). Data on patient race and ethnicity were obtained from the EHRs and were evaluated in this study to characterize the patient population receiving care in the participating sites.

### Clinician and Staff Participants

All clinicians, including prescribing (ie, physicians, nurse practitioners, and physician assistants) and nonprescribing (eg, psychologists and social workers) clinicians, as well as staff (eg, nurses and community health workers) who had been employed at the given site for 6 months or longer, were invited to complete a survey at study initiation and then every 6 months for a total of 6 follow-up surveys. Responses from individuals who did not have a role involving provision of clinical services (ie, administrative staff or data coordinator) and/or were missing all responses on outcomes of interest (ie, readiness rulers and Organizational Readiness to Change Assessment [ORCA]^19^) on relevant surveys were excluded. The decision to complete the survey was considered consent to study participation.

### Randomization and Blinding

Given concerns for potential contamination by a different National Institute on Drug Abuse–funded project at 1 of the sites (which was not ultimately implemented at this site), 1 site was assigned to receive facilitation last. The remaining 3 sites were randomized by the statistician to the date when facilitation would begin at their site. Members of the investigative team and study sites remained blinded to the sequence until approximately 6 weeks before the start of facilitation to allow for planning activities.

### Procedures

Informed by prior efforts to promote integration of mental health treatment into primary care,^6^ the approach and details of our manualized facilitation have been published previously.^13^ Facilitation started with a baseline mixed-methods formative evaluation of barriers and facilitators to promoting addiction treatments in HIV clinics.^14^ The external facilitators, including a team of 4 physicians (E.J.E., K.L.M., S.B.M., and D.A.F.) with expertise in addiction medicine, addiction psychiatry, and/or HIV, worked with each of the sites to identify local champions and promote site engagement. Then during 2 follow-up visits to each site over the 6-month facilitation period, the external facilitators (E.J.E., K.L.M., S.B.M., and D.A.F.) conducted academic detailing and facilitated networking opportunities across disciplines within the same institution with the goal of building relationships and training opportunities. The external facilitators also had ongoing communications (via email and telephone) with the sites to facilitate additional facilitation activities. Upon initiation of the facilitation period, sites were invited to join learning collaborative activities, which included a monthly webinar with a mix of didactics and case-based learning and receipt of a monthly newsletter with resources (eg, journal articles, addiction-focused scientific conferences, and training opportunities). Sites were encouraged to conduct program marketing (eg, pens, pads, posters, and pins with the phrase “WHAT IF?” designed to engage patients and clinicians in a conversation about substance use) and to develop processes for performance monitoring and feedback, and they were provided site-specific data on prevalence of OUD, AUD, and TUD based on the EHR data and rates of treatment at 2 time points. After crossing over from the control period to 6-month facilitation, sites were then considered to be in the 6-month evaluation period, followed by the maintenance period that lasted the duration of the study. Before facilitation onset and then every 6 months thereafter for the duration of the study, EHR data were extracted and confidential web-based Qualtrics surveys were administered.

### Outcomes

Implementation outcomes included change in the proportion of patients with one of the 3 targeted SUDs who received MAT during the evaluation (primary) and maintenance periods compared with the control period. We specifically examined receipt of MAT, measured using EHR data, that may be prescribed through HIV clinics (eTable 1 in Supplement 2) and provision of counseling. A patient was considered to have an active prescription in a given 6-month study period if they had medication coverage during the period of interest based on the days supplied and assuming the medication was taken as prescribed; for injectable naltrexone, we assumed coverage lasted for 30 days and was administered on schedule as prescribed. In secondary analyses, we also assessed provision of counseling as documented on the basis of encounters with a clinician, social worker, or psychologist and including psychiatric and substance use assessments, individual and group psychotherapy, individual counseling, case management, crisis intervention, prolonged services, family services, and health and behavior education.

Additional secondary implementation outcomes included clinician, staff, and organizational readiness to promote MAT and counseling for OUD, AUD and TUD. Clinician and staff readiness were measured on a readiness ruler (eg, “How ready are you to prescribe or refer patients for medications [i.e., nicotine replacement therapy, bupropion, and varenicline] for the treatment of tobacco use disorder?”), where response options ranged from 0 (not ready) to 10 (ready) on a continuous scale. This assessment was collected during all survey waves except when inadvertently not collected during 1 period (July 26, 2019, to January 25, 2020).

Organizational readiness was assessed with a modified ORCA^19^ with which participants were asked to rate the evidence supporting each evidence-based practice and the context as a setting for delivering addiction treatments. Subscale response options also included a 5-point Likert scale, ranging from 1 (very infrequently) to 5 (very frequently). Subscale response options also included do not know or not applicable, which were recoded as neither agree nor disagree or neither frequently nor infrequently to allow computation of subscale scores.^20^

### Statistical Analysis

On the basis of prior work,^7,21,22,23^ we hypothesized we would detect an 11% and 19% absolute increase in provision of MAT during the evaluation period and maintenance period, respectively, compared with the control period. Accounting for the stepped wedge design with a cross-sectional analytic approach with 4 steps of 6 months each, 1 baseline measurement, and an intraclass correlation coefficient of 0.01,^24^ we estimated that a sample size of 375 across the 4 clinics would be necessary to detect these differences with at least 90% power and a type I error rate of 5%.

We used descriptive statistics to characterize the baseline characteristics of the clinic populations. For all analyses, we used an intention-to-treat approach based on the time clinics were intended to cross over from control condition to facilitation. For the primary implementation outcomes and other measurements in this study, including readiness to provide MAT, ORCA evidence ratings for MAT, and ORCA context ratings for MAT, we used generalized estimating equation models with study phase, site, and natural time to generate adjusted odds ratios or mean differences and associated 95% CIs measuring the effect of facilitation compared with the control period at each study period. Compound symmetry working correlation matrix was specified to control for correlation of repeated measures within subjects. In secondary analyses, we assessed provision of MAT with counseling. In sensitivity analyses, we included all clinic patients regardless of SUD diagnosis given concerns that SUD diagnoses may not be uniformly captured and separately reran primary analyses excluding the final study period when the first wave of COVID-19 pandemic started (January 26 to July 25, 2020). We applied a similar approach to describe clinician and staff participants and then evaluated the impact of facilitation on the readiness ruler and ORCA subscale scores. Two-sided *P* < .05 was considered significant. All analyses were performed using SAS statistical software version 9.4 (SAS Institute). Data analysis was performed from August 2020 to September 2022.

## Results

### Clinic Patient Populations

At study start, a total of 3647 patients were engaged in care across the 4 clinics (range, 366-1548 patients per clinic). Among 3647 patients, the mean (SD) age was 49 (12) years, 1814 (50%) were Black, 781 (22%) were Hispanic, and 1407 (39%) were female; 121 (3%) had opioid use disorder, 126 (3%) had alcohol use disorder, and 420 (12%) had tobacco use disorder (Table 1).

**Table 1.** Baseline Patient Characteristics by Substance Use Disorder

Characteristic	Patients, No. (%)			
	Opioid use disorder (n = 121)	Alcohol use disorder (n = 126)	Tobacco use disorder (n = 420)	Total (N = 3647)
Age, mean (SD), y	52 (9)	50 (11)	51 (11)	49 (12)
Race				
Asian	0	1 (1)	0	19 (1)
Black	39 (32)	61 (48)	219 (52)	1814 (50)
White	46 (38)	42 (33)	121 (29)	1118 (31)
Other^a^	36 (30)	22 (18)	80 (19)	689 (19)
Missing^b^	0	0	0	7
Ethnicity				
Hispanic	39 (32)	34 (27)	87 (21)	781 (22)
Non-Hispanic	82 (68)	92 (73)	333 (79)	2859 (79)
Missing^b^	0	0	0	7
Sex				
Female	41 (34)	31 (25)	161 (38)	1407 (39)
Male	80 (66)	95 (75)	259 (62)	2240 (61)
Public insurance				
Yes	47 (61)	69 (81)	252 (81)	1725 (70)
No	30 (39)	16 (19)	61 (20)	740 (30)
Missing^b^	44	41	107	1182
Self-pay				
Yes	0	0	5 (2)	21 (1)
No	77 (100)	85 (100)	308 (98)	2444 (99)
Missing^b^	44	41	107	1182
Private or commercial insurance				
Yes	35 (46)	29 (34)	89 (28)	985 (40)
No	42 (55)	56 (66)	224 (72)	1480 (60)
Missing^b^	44	41	107	1182
Income in the ZIP code, $				
Median (range)	68 035 (59 805-98 187)	68 035 (45 063-98 187)	62 276 (45 063-110 485)	62 276 (45 063-172 243)
Missing^b^	0	0	0	2
Completed visits, median (range), No.	3 (0-14)	2 (0-15)	2 (0-13)	2 (0-17)
Prescribed antiretroviral therapy				
Yes	115 (95)	119 (94)	404 (96)	3430 (94)
No	6 (5)	7 (6)	16 (4)	217 (6)
Detectable HIV viral load (>200 copies/mL)				
Yes	16 (16)	19 (18)	43 (11)	325 (11)
No	83 (84)	84 (82)	349 (89)	2774 (90)
Missing^b^	22	23	28	548
Hepatitis C virus infection				
Yes	70 (59)	40 (32)	110 (26)	522 (15)
No	49 (41)	86 (68)	307 (74)	3072 (86)
Missing^b^	2	0	3	53
CD4 cell count, cells/μL				
<50	0	0	2 (1)	31 (1)
50-99	3 (4)	2 (2)	7 (2)	42 (2)
100-199	9 (11)	10 (11)	27 (8)	154 (6)
200-349	14 (17)	16 (18)	39 (11)	336 (12)
350-499	19 (22)	21 (23)	54 (15)	480 (18)
>500	40 (47)	42 (46)	225 (64)	1697 (62)
Missing^b^	36	35	66	907

^a^ Other refers to American Indian or Alaska Native, Native Hawaiian or other Pacific Islander, or any other race not specified.

^b^ Missing data were not included in calculations of percentages.

### Impact of Facilitation on Provision of MAT Alone and With Counseling

**Figure 1.**
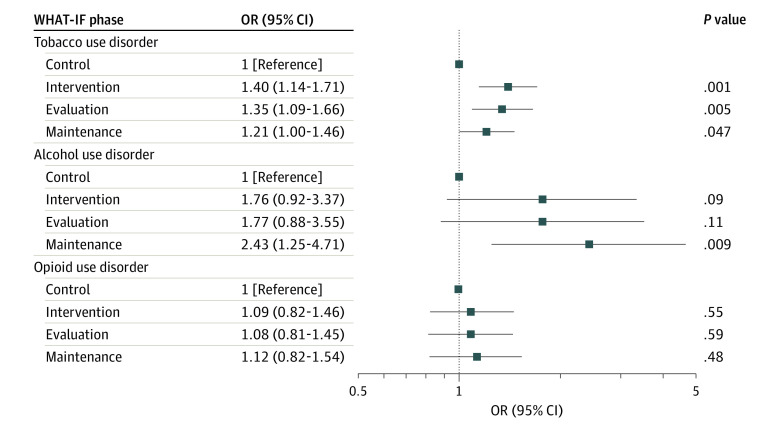
Provision of Medications for Addiction Treatment Among Treatment-Eligible Patients Across Sites by Study Period OR indicates odds ratio; WHAT-IF, Working with HIV Clinics to adopt Addiction Treatment using Implementation Facilitation.

Among patients with OUD, compared with the control period (243 patients [27%; 95% CI, 22%-32%]), we did not observe an increase in provision of MOUD with facilitation during the evaluation period (135 patients [28%; 95% CI, 22%-35%]; *P* = .59) or maintenance period (198 patients [29%; 95% CI, 22%-36%]; *P* = .48) (Table 2 and Figure 1). Among patients with AUD, compared with the control period (251 patients [8%; 95% CI, 5%-12%]), there was an increase in provision of MAUD with facilitation during the evaluation period, although the difference was not significant (112 patients [13%; 95% CI, 8%-21%]; *P* = .11); however, the difference from the control period increased and became significant during the maintenance period (180 patients [17%; 95% CI, 12%-24%]; *P* = .009) (Table 2 and Figure 1). Among patients with TUD, compared with the control period (810 patients [33%; 95% CI, 30%-36%]), we observed significant increases in provision of MTUD with facilitation during both the evaluation (471 patients [40%; 95% CI, 36%-45%; *P* = .005) and maintenance (643 patients [38%; 95% CI, 34%-41%]; *P* = .047) periods (Table 2 and Figure 1). The findings were not substantially different in secondary analyses focused on MAT with counseling, with sensitivity analyses including all clinic patients regardless of the presence of a SUD diagnosis or when excluding the period impacted by COVID-19 .

**Table 2.** Provision of Medications for Addiction Treatment Among Treatment-Eligible Patients Across All Sites by Study Period, Results From Generalized Estimating Equation

Study period	Provision of MOUD^a^		Provision of MAUD		Provision of MTUD	
	Patients, No. (%) [95% CI]	*P* value	Patients, No. (%) [95% CI]	*P* value	Patients, No. (%) [95% CI]	*P* value
Control	243 (27) [22-32]	Reference	251 (8) [5-12]	Reference	810 (33) [30-36]	Reference
Intervention	117 (28) [22-35]	.55	122 (13) [8-21]	.09	444 (41) [37-46]	.001
Evaluation	135 (28) [22-35]	.59	112 (13) [8-21]	.11	471 (40) [36-45]	.005
Maintenance	198 (29) [22-36]	.48	180 (17) [12-24]	.009	643 (38) [34-41]	.047

Abbreviations: MAUD, medications for alcohol use disorder; MOUD, medications for opioid use disorder; MTUD, medications for tobacco use disorder.

^a^ MOUD exclusively included buprenorphine products.

### Clinician and Staff Populations

Among 131 invited participants, 85 completed the baseline survey (65% response rate). We excluded 8 administrative staff and 7 with missing data on all readiness rulers and ORCA subscales. Clinician and staff participant characteristics are reported in Table 3.

**Table 3.** Baseline Clinician and Staff Participant Characteristics

Characteristic	Participants, No. (%)				
	Site A (n = 24)	Site B (n = 11)	Site C (n = 12)	Site D (n = 23)	Total (N = 70)
Age, mean (SD), y	63 (54)	57 (5)	48 (10)	60 (46)	58 (41)
Sex					
Female	16 (67)	6 (55)	10 (83)	19 (83)	51 (73)
Male	8 (33)	5 (45)	2 (17)	4 (17)	19 (27)
Race					
Asian	2 (9)	2 (18)	1 (8)	1 (4)	6 (9)
Black	7 (30)	2 (18)	0	1 (4)	10 (14)
White	12 (52)	7 (64)	7 (58)	19 (83)	45 (65)
Other^a^	2 (9)	0	4 (33)	2 (9)	8 (12)
Ethnicity					
Hispanic	3 (13)	0	7 (58)	2 (9)	12 (17)
Non-Hispanic	17 (74)	10 (91)	5 (42)	20 (87)	52 (75)
Other^a^	3 (13)	1 (9)	0	1 (4)	5 (7)
Missing^b^	1	0	0	0	1
Clinician (physicians, physician assistant, nurse practitioner)					
Yes	11 (46)	7 (64)	4 (33)	12 (52)	34 (49)
No	13 (54)	4 (36)	8 (67)	11 (48)	36 (51)
Time working at this clinic, mean (SD), y	5 (5)	12 (10)	7 (8)	7 (8)	7 (8)
Time per week spent working at HIV clinic, median (range), h	25 (3-55)	40 (4-40)	36 (12-40)	18 (4-50)	31 (3-55)
Ever prescribed medications to treat tobacco use disorder (ie, nicotine replacement therapy, bupropion, varenicline), yes	9 (82)	7 (100)	4 (100)	12 (100)	32 (94)
Ever provided counseling to treat tobacco use disorder, yes	11 (100)	7 (100)	4 (100)	12 (100)	34 (100)
Ever prescribed medications to treat unhealthy alcohol use (ie, disulfiram, acamprosate, oral or injectable naltrexone, other), yes	3 (27)	3 (43)	1 (25)	7 (58)	14 (41)
Ever provided counseling to treat unhealthy alcohol use, yes	11 (100)	7 (100)	4 (100)	12 (100)	34 (100)
Ever provided counseling to treat opioid use disorder, yes	9 (82)	7 (100)	4 (100)	12 (100)	32 (94)
Hold a waiver that allows buprenorphine (eg, Suboxone) prescribing, yes	4 (36)	1 (14)	1 (25)	5 (42)	11 (32)
Ever prescribed oral or injectable (eg, Vivitrol) naltrexone to treat opioid use disorder, yes	1 (9)	1 (14)	1 (25)	2 (17)	5 (15)

^a^ Other refers to American Indian or Alaska Native, Native Hawaiian or Pacific Islander, or any other race not specified.

^b^ Missing data were not included in calculations of percentages.

### Clinician, Staff, and Organizational Readiness to Provide MAT

**Figure 2.**
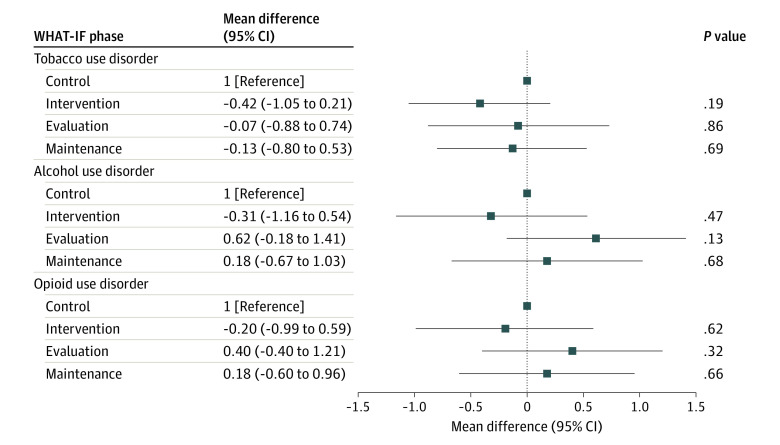
Clinician and Staff Readiness Across All Sites to Provide Medications for Addiction Treatment by Study Period WHAT-IF indicates Working with HIV Clinics to adopt Addiction Treatment using Implementation Facilitation.

Compared with the control period, we did not observe an increase in readiness to provide MOUD, MAUD, or MTUD with facilitation during the evaluation or maintenance periods (Figure 2 and eTable 2 in Supplement 2). Compared with the control period, we observed an increase in evidence subscale scores for MAUD with facilitation during the maintenance period; we did not observe any other changes during the evaluation or maintenance periods otherwise (eTable 3 and eFigure 2 in Supplement 2). Similarly, we did not observe any changes in the context subscale scores over the study periods (eTable 4 and eFigure 3 in Supplement 2).

## Discussion

To our knowledge, this randomized clinical trial is the first study to evaluate the impact of facilitation on promoting evidence-based addiction treatment to address OUD, AUD, and TUD in HIV clinics, and it generated several key findings. First, facilitation yielded improvements in clinician and staff self-rated readiness to provide MAUD and a corresponding increase in provision of these medications. Second, facilitation was not sufficient to result in measurable or consistent changes in readiness or actual provision of MOUD. Third, facilitation resulted in improvements in provision of MTUD without measurable change in clinician or staff readiness in the context of high baseline readiness. Fourth, clinician and staff consistently reported high readiness to provide MAT for these life-threatening conditions, but this did not translate into actual provision of these medications. Our findings suggest that facilitation as implemented, with a primary focus on clinician and staff-level factors, was insufficient for promoting high-levels of provision of MOUD, MAUD, and MTUD in HIV clinics.

Prior studies^7,8^ have demonstrated benefits of facilitation on promoting chronic disease management in general medical settings, yet these studies have generally not targeted addiction treatment. Instead, previous studies to promote addiction treatment have focused on evaluating academic detailing (a potential component of facilitation or stand-alone intervention) to improve treatment of a specific use disorder and demonstrated greatest benefits in the context of low baseline prescribing and high density of treatment-eligible patients.^10,11^ Our study extends this literature by applying facilitation in HIV clinics to simultaneously promote provision of treatment of the 3 SUDs for which effective medication and behavioral interventions are available.

Our findings that facilitation resulted in increased provision of MTUD, delayed improvements in MAUD, and no observed improvements in MOUD correspond with observed patterns in clinician and staff readiness. In the context of high baseline clinician and staff readiness to prescribe MTUD treatment, with some focused clinician education and academic detailing coupled with clinic-level processes stimulated by facilitation (eg, nurse-led protocols), it was possible to change practices to promote MTUD in a short time. On the other hand, the observed delayed increases in MAUD may be explained by the fact that higher levels of education and academic detailing (ie, more interactions) may be required to enhance clinician and staff readiness, particularly in the absence of a local champion. Finally, the fact that we did not observe increases in clinician and staff readiness to provide MOUD or increased provision of MOUD may be explained, at least in part, by the fact that all sites had at least 1 clinician who prescribed buprenorphine at the time the study was initiated, perhaps contributing to a lower perceived need and sufficient MOUD services.

Although it is encouraging that facilitation resulted in some increases in provision of MAT, our findings suggest that additional strategies may be needed. First, facilitation may have been more effective by sequentially focusing on different use disorders rather than 3 simultaneously. Second, facilitation may have been more effective if provided over a longer time or with higher intensity of contacts and support. Third, clinician and staff may have benefited from further training in motivational interviewing techniques to engage patients in care. Finally, efforts to more directly link provision of MAT to reimbursement may be required to help prioritize MAT in these settings given the multiple, complex existing demands.^25^

### Limitations and Strengths

Our study has limitations. First, our primary outcome focused on provision of any treatment during a given period among treatment-eligible patients within the given clinic; we did not distinguish between treatment initiation and continuation or assess treatment duration, nor were we focused on medications that may have been provided elsewhere (eg, opioid treatment programs). Second, treatment eligibility relied on clinician coding of SUD, which undercounts true prevalence.^26^ Third, we are unable to determine whether counseling as measured in the EHR was specifically provided to address a given SUD. Fourth, self-reported outcomes are subject to social desirability bias, survey fatigue, and assessment reactivity.^27^ Fifth, we had missing data on readiness scales during 1 period. Sixth, our study was conducted in HIV clinicals all located in the northeastern US and thus may not be generalizable to other settings. Given their willingness to participate in a study focused on promoting addiction treatment, these sites may have had higher baseline readiness to provide addiction treatment than the typical HIV clinic. Seventh, our findings may have been impacted by temporal trends, a time when there has been greater focus on enhancing treatment of OUD, and also threatened by the COVID-19 pandemic.

Our study also has important strengths. First, for our primary outcome, we relied on EHR data to assess MAT provision that were routinely collected, thus minimizing selection bias and allowing for robust ascertainment of our primary outcome.^28^ Second, our study was conducted in both community and academically affiliated HIV clinics with varying levels of resources and infrastructure to enhance generalizability. Third, with the exception of 1 site, site personnel and study investigators were blinded as to when each site would receive the intervention to minimize prefacilitation activities.

## Conclusions

In this randomized clinical trial, facilitation resulted in improvements in MTUD and MAUD with no measurable change in MOUD provision. Given the importance of these treatments to people with HIV and observed treatment gaps, robust implementation strategies are needed to reach individuals with HIV engaged in care.

## Notes

Supplement 1.
Trial Protocol

Supplement 2. **eFigure 1.** CONSORT Flow Diagram **eTable 1.** Medications Available for HIV Clinic-Based Addiction Treatment by Substance Use Disorder **eTable 2.** Clinician and Staff Readiness to Provide MAT by Study Period, Results From GEE **eTable 3.** Clinician and Staff ORCA Evidence Ratings for MAT by Study Period, Results From GEE **eFigure 2.** Clinician and Staff ORCA Evidence Ratings for Medications for Addiction Treatment by Study Period **eTable 4.** Clinician and Staff ORCA Context Ratings for MAT by Study Period, Results From GEE **eFigure 3.** Clinician and Staff ORCA Context Ratings for Medications for Addiction Treatment by Study Period

Supplement 3.
Data Sharing Statement

## References

[1] Department of Health and Human Services. Guidelines for the use of antiretroviral agents in adults and adolescents with HIV. February 2021. Accessed September 12, 2022. https://health.gov/healthypeople/tools-action/browse-evidence-based-resources/guidelines-use-antiretroviral-agents-adults-and-adolescents-living-hiv

[2] Oldfield BJ, McGinnis KA, Edelman EJ, . Predictors of initiation of and retention on medications for alcohol use disorder among people living with and without HIV. J Subst Abuse Treat. 2020;109:14-22. doi:10.1016/j.jsat.2019.11.002 31856946 PMC6982467

[3] Shahrir S, Crothers K, McGinnis KA, . Receipt and predictors of smoking cessation pharmacotherapy among veterans with and without HIV. Prog Cardiovasc Dis. 2020;63(2):118-124. doi:10.1016/j.pcad.2020.01.003 31987807 PMC7251937

[4] Chander G, Monroe AK, Crane HM, . HIV primary care providers: screening, knowledge, attitudes and behaviors related to alcohol interventions. Drug Alcohol Depend. 2016;161:59-66. doi:10.1016/j.drugalcdep.2016.01.015 26857898 PMC4841449

[5] Bold KW, Deng Y, Dziura J, . Practices, attitudes, and confidence related to tobacco treatment interventions in HIV clinics: a multisite cross-sectional survey. Transl Behav Med. 2022;12(6):726-733. doi:10.1093/tbm/ibac022 35608982 PMC9260059

[6] Ritchie MJ, Dollar KM, Miller CJ, . Using implementation facilitation to improve care in the Veterans Health Administration (version 2). 2017. Accessed September 12, 2022. https://www.queri.research.va.gov/tools/Facilitation-Manual.pdf

[7] Baskerville NB, Liddy C, Hogg W. Systematic review and meta-analysis of practice facilitation within primary care settings. Ann Fam Med. 2012;10(1):63-74. doi:10.1370/afm.1312 22230833 PMC3262473

[8] Wang A, Pollack T, Kadziel LA, . Impact of practice facilitation in primary care on chronic disease care processes and outcomes: a systematic review. J Gen Intern Med. 2018;33(11):1968-1977. doi:10.1007/s11606-018-4581-9 30066117 PMC6206351

[9] D’Onofrio G, Edelman EJ, Hawk KF, . Implementation facilitation to promote emergency department-initiated buprenorphine for opioid use disorder: protocol for a hybrid type III effectiveness-implementation study (Project ED HEALTH). Implement Sci. 2019;14(1):48. doi:10.1186/s13012-019-0891-5 31064390 PMC6505286

[10] Williams EC, Matson TE, Harris AHS. Strategies to increase implementation of pharmacotherapy for alcohol use disorders: a structured review of care delivery and implementation interventions. Addict Sci Clin Pract. 2019;14(1):6. doi:10.1186/s13722-019-0134-8 30744686 PMC6371480

[11] Leone FT, Evers-Casey S, Graden S, Schnoll R, Mallya G. Academic detailing interventions improve tobacco use treatment among physicians working in underserved communities. Ann Am Thorac Soc. 2015;12(6):854-858. doi:10.1513/AnnalsATS.201410-466BC 25867533 PMC4590019

[12] NIH Research Portfolio Online Reporting Tools (RePORT). Implementation to motivate physician response to opioid dependence in HIV setting. Accessed September 7, 2021. https://reporter.nih.gov/search/Z9RxTlyRv0yPQ__0ft3Kkg/project-details/9656986#publications

[13] Edelman EJ, Dziura J, Esserman D, . Working with HIV clinics to adopt addiction treatment using implementation facilitation (WHAT-IF?): rationale and design for a hybrid type 3 effectiveness-implementation study. Contemp Clin Trials. 2020;98:106156. doi:10.1016/j.cct.2020.106156 32976995 PMC7511156

[14] Edelman EJ, Gan G, Dziura J, . Readiness to provide medications for opioid, alcohol and tobacco use disorder in HIV clinics: a multi-site mixed-methods formative evaluation. J Acquir Immune Defic Syndr. 2021;87(3):959-970. doi:10.1097/QAI.0000000000002666 33675619 PMC8192340

[15] Curran GM, Bauer M, Mittman B, Pyne JM, Stetler C. Effectiveness-implementation hybrid designs: combining elements of clinical effectiveness and implementation research to enhance public health impact. Med Care. 2012;50(3):217-226. doi:10.1097/MLR.0b013e3182408812 22310560 PMC3731143

[16] Beard E, Lewis JJ, Copas A, . Stepped wedge randomised controlled trials: systematic review of studies published between 2010 and 2014. Trials. 2015;16:353. doi:10.1186/s13063-015-0839-2 26278881 PMC4538902

[17] Schulz KF, Altman DG, Moher D; CONSORT Group. CONSORT 2010 statement: updated guidelines for reporting parallel group randomized trials. Ann Intern Med. 2010;152(11):726-732. doi:10.7326/0003-4819-152-11-201006010-00232 20335313

[18] van den Berg JJ, O’Keefe E, Davidson D, . The development and evaluation of an HIV implementation science network in New England: lessons learned. Implement Sci Commun. 2021;2(1):64. doi:10.1186/s43058-021-00165-2 34112269 PMC8192037

[19] Helfrich CD, Li YF, Sharp ND, Sales AE. Organizational readiness to change assessment (ORCA): development of an instrument based on the Promoting Action on Research in Health Services (PARIHS) framework. Implement Sci. 2009;4:38. doi:10.1186/1748-5908-4-38 19594942 PMC2716295

[20] Hawk KF, D’Onofrio G, Chawarski MC, . Barriers and facilitators to clinician readiness to provide emergency department–initiated buprenorphine. JAMA Netw Open. 2020;3(5):e204561. doi:10.1001/jamanetworkopen.2020.4561 32391893 PMC7215257

[21] Garner BR. Research on the diffusion of evidence-based treatments within substance abuse treatment: a systematic review. J Subst Abuse Treat. 2009;36(4):376-399. doi:10.1016/j.jsat.2008.08.004 19008068 PMC2695403

[22] Knudsen HK, Ducharme LJ, Roman PM. Early adoption of buprenorphine in substance abuse treatment centers: data from the private and public sectors. J Subst Abuse Treat. 2006;30(4):363-373. doi:10.1016/j.jsat.2006.03.013 16716852

[23] Kirchner JE, Ritchie MJ, Pitcock JA, Parker LE, Curran GM, Fortney JC. Outcomes of a partnered facilitation strategy to implement primary care-mental health. J Gen Intern Med. 2014;29(suppl 4):904-912. doi:10.1007/s11606-014-3027-2 25355087 PMC4239280

[24] Woertman W, de Hoop E, Moerbeek M, Zuidema SU, Gerritsen DL, Teerenstra S. Stepped wedge designs could reduce the required sample size in cluster randomized trials. J Clin Epidemiol. 2013;66(7):752-758. doi:10.1016/j.jclinepi.2013.01.009 23523551

[25] HRSA. HIV/AIDS Bureau performance measures. November 2, 2019. Accessed September 12, 2022. https://hab.hrsa.gov/sites/default/files/hab/clinical-quality-management/adolescentadultmeasures.pdf

[26] Holt SR, Ramos J, Harma M, . Physician detection of unhealthy substance use on inpatient teaching and hospitalist medical services. Am J Drug Alcohol Abuse. 2013;39(2):121-129. doi:10.3109/00952990.2012.715703 22992028

[27] Heishman SJ, Saha S, Singleton EG. Imagery-induced tobacco craving: duration and lack of assessment reactivity bias. Psychol Addict Behav. 2004;18(3):284-288. doi:10.1037/0893-164X.18.3.284 15482084

[28] Hemming K, Taljaard M, Grimshaw J. Introducing the new CONSORT extension for stepped-wedge cluster randomised trials. Trials. 2019;20(1):68. doi:10.1186/s13063-018-3116-3 30658677 PMC6339370

