# Supplementary material for: Effect of Implementation Facilitation to Promote Adoption of Medications for Addiction Treatment in US HIV Clinics: A Randomized Clinical Trial
Source: JAMA Netw Open. 2022 Oct 17;5(10):e2236904. doi: 10.1001/jamanetworkopen.2022.36904 (PMC9577676; doi:10.1001/jamanetworkopen.2022.36904)
Supplement: Supplement 1. — Trial Protocol [file jamanetwopen-e2236904-s001.pdf]

YALE UNIVERSITY
HUMAN INVESTIGATION COMMITTEE

Application to Involve Human Subjects in Biomedical Research
100 FR1 (2015-1)

SECTION I: ADMINISTRATIVE INFORMATION

<table>
<tr><td colspan="4">Title of Research Project:
Working with HIV clinics to adopt addiction treatments using implementation facilitation (WHAT IF?).</td></tr>
<tr><td colspan="2">Principal Investigator:
David Fiellin, MD</td><td colspan="2">Yale Academic Appointment:
Professor</td></tr>
<tr><td colspan="4">Department: Internal Medicine</td></tr>
<tr><td colspan="4">Campus Address:
367 Cedar Street, Suite 401 A, New Haven, CT 06510</td></tr>
<tr><td>Campus Phone: 203-737-3347</td><td>Fax:203-737-3306</td><td>Pager:860-588-4505</td><td>E-mail: david.fiellin@yale.edu</td></tr>
<tr><td colspan="4">Protocol Correspondent Name & Address (if different than PI):</td></tr>
<tr><td>Campus Phone:</td><td></td><td colspan="2">E-mail:</td></tr>
<tr><td colspan="4">Yale Cancer Center CTO Protocol Correspondent Name & Address (if applicable):</td></tr>
<tr><td>Campus Phone:</td><td>Fax:</td><td colspan="2">E-mail:</td></tr>
<tr><td colspan="4">Business Manager:</td></tr>
<tr><td>Campus Phone :</td><td>Fax :</td><td colspan="2">E-mail</td></tr>
</table>

<table>
<tr><td colspan="2">Faculty Advisor:(required if PI is a student, resident, fellow or other trainee) ☒ NA</td><td colspan="2">Yale Academic Appointment:</td></tr>
<tr><td colspan="4">Campus Address:</td></tr>
<tr><td>Campus Phone:</td><td>Fax:</td><td>Pager:</td><td>E-mail:</td></tr>
</table>

Investigator Interests:

Does the principal investigator, or do any research personnel who are responsible for the design, conduct or reporting of this project or any of their family members (spouse or dependent child) have an incentive or interest, financial or otherwise, that may affect the protection of the human subjects involved in this project, the scientific objectivity of the research or its integrity? Note: The Principal Investigator (Project Director), upon consideration of the individual’s role and degree of independence in carrying out the work, will determine who is responsible for the design, conduct, or reporting of the research.

See Disclosures and Management of Personal Interests in Human Research
http://www.yale.edu/hrpp/policies/index.html#COI
o Yes X No

Do you or does anyone on the research team who is determined by you to be responsible for the design, conduct or reporting of this research have any patent (sole right to make, use or sell an invention) or copyright (exclusive rights to an original work) interests related to this research protocol?
o Yes X No

If yes to either question above, list names of the investigator or responsible person:

The Yale University Principal Investigator, all Yale University co-investigators, and all Yale University individuals who are responsible for the design, conduct or reporting of research must have a current financial disclosure form on file with the University's Conflict of Interest Office. Yale New Haven Hospital personnel who are listed as co-investigators on a protocol with a Yale University Principal Investigator must also have a current financial disclosure form on file with the University's Conflict of Interest Office. If this has not been done, the individual(s) should follow this link to the COI Office Website to complete the form: http://www.yale.edu/coi/

NOTE: The requirement for maintaining a current disclosure form on file with the University's Conflict of Interest Office extends primarily to Yale University and Yale-New Haven Hospital personnel. **Whether or not they are required to maintain a disclosure form with the University's Conflict of Interest Office, all investigators and individuals deemed otherwise responsible by the PI who are listed on the protocol are required to disclose to the PI any interests that are specific to this protocol.**

SECTION II: GENERAL INFORMATION

1. **Performing Organizations:** Identify the hospital, in-patient or outpatient facility, school or other agency that will serve as the location of the research. Choose all that apply:

a. Internal Location[s] of the Study:

☐ Magnetic Resonance Research Center (MR-TAC)
☐ Yale Cancer Center/Clinical Trials Office (CTO)
☐ Yale Cancer Center/Smilow
☒ Yale-New Haven Hospital - Nathan Smith Clinic
☒ Yale-New Haven Hospital—Saint Raphael Campus – Haelen Center
☐ Cancer Data Repository/Tumor Registry
☐ Specify Other Yale Location:
☐ Yale University PET Center
☐ YCCI/Church Street Research Unit (CSRU)
☐ YCCI/Hospital Research Unit (HRU)
☐ YCCI/Keck Laboratories

b. External Location[s]:

☐ APT Foundation, Inc.
☐ Connecticut Mental Health Center
☐ Haskins Laboratories
☐ John B. Pierce Laboratory, Inc.

☐ Clinical Neuroscience Research Unit (CNRU) ☐ Veterans Affairs Hospital, West Haven
☒ Other Locations, Specify: ☐ International Research Site
(Specify location(s)):

Immunology Center at Miriam Hospital in Providence, RI; Hartford Hospital's HIV Clinic in Hartford, CT, and SUNY Downstate Medical Center's HIV clinic in Brooklyn, NY.

c. Additional Required Documents *(check all that apply)*: ☒ N/A

☐ *YCCI-Scientific and Safety Committee (YCCI-SSC) Approval Date:
☐ *Pediatric Protocol Review Committee (PPRC) Approval Date:
☐ *YCC Protocol Review Committee (YRC-PRC) Approval Date:
☐ *Dept. of Veterans Affairs, West Haven VA HSS Approval Date:
☐ *Radioactive Drug Research Committee (RDRC) Approval Date:
☐ YNHH-Radiation Safety Committee (YNHH-RSC) Approval Date:
☐ Magnetic Resonance Research Center PRC (MRRC-PRC) Approval Date:
☐ YSM/YNHH Cancer Data Repository (CaDR) Approval Date:
☐ Dept. of Lab Medicine request for services or specimens form
☐ Imaging on YNHH Diagnostic Radiology equipment request form (YDRCTO request) found at http://radiology.yale.edu/research/ClinTrials.aspx)

Approval from these committees is required before final HIC approval is granted. See instructions for documents required for initial submission and approval of the protocol. Allow sufficient time for these requests. Check with the oversight body for their time requirements.

2. **Probable Duration of Project:** State the expected duration of the project, including all follow-up and data analysis activities. **7 years**

3. **Research Type/Phase: (Check all that apply)**

a. **Study Type**

☐ Single Center Study
☒ Multi-Center Study

Does the Yale PI serve as the PI of the multi-site study? Yes ☒ No ☐

☒ Coordinating Center/Data Management
☐ Other:

b. **Study Phase** ☐ **N/A**

☐ Pilot ☐ Phase I ☐ Phase II ☐ Phase III ☒ Phase IV
☐ Other (*Specify)*

4. **Area of Research: (Check all that apply)** Note that these are overlapping definitions and more than one category may apply to your research protocol. Definitions for the following can be found in the instructions section 4c:

☒ Clinical Research: Patient-Oriented
☐ Clinical Research: Epidemiologic and Behavioral
☐ Translational Research #1 ("Bench-to-Bedside")
☒ Translational Research #2 ("Bedside-to-Community")
☒ Clinical Research: Outcomes and Health Services
☐ Interdisciplinary Research
☐ Community-Based Research

5. Is this study a clinical trial? Yes ☒ No ☐

NOTE the current ICMJE (International Committee of Medical Journal Editors) definition of a clinical trial: "any research study that prospectively assigns human participants or groups of humans to one or more health-related interventions to evaluate the effects on health outcomes." Health-related interventions include any intervention used to modify a biomedical or health-related outcome (for example, drugs, surgical procedures, devices, behavioral treatments, dietary interventions, and process-of-care changes). Health outcomes include any biomedical or health-related measures obtained in patients or participants, including pharmacokinetic measures and adverse events"

If yes, where is it registered?

Clinical Trials.gov registry ☒

Other (*Specify*)

Registration of clinical trials ***at their initiation*** *is required by the FDA, NIH and by the ICMJE.*

If this study is registered on clinicaltrials.gov, there is new language in the consent form and compound authorization that should be used.

For more information on registering clinical trials, including whether your trial must be registered, see the YCCI webpage, http://ycci.yale.edu/researchers/ors/registerstudy.aspx or contact YCCI at 203.785.3482)

6. Does the Clinical Trials Agreement (CTA) require compliance with ICH GCP (E6)?
Yes ☐ No☒

7. Will this study have a billable service? *A billable service is defined as any service rendered to a study subject that, if he/she was not on a study, would normally generate a bill from either Yale-New Haven Hospital or Yale Medical Group to the patient or the patient's insurer. The service may or may not be performed by the research staff on your study, but may be provided by professionals within either Yale-New Haven Hospital or Yale Medical Group (examples include x-rays, MRIs, CT scans, specimens sent to central labs, or specimens sent to pathology). Notes: 1. There is no distinction made whether the service is paid for by the subject or their insurance (Standard of Care) or by the study's funding mechanism (Research Sponsored). 2. This generally includes new services or orders placed in EPIC for research subjects.*

Yes ☐ No☒

If answered, "yes", this study will need to be set up in OnCore, Yale's clinical research management system, for Epic to appropriately route research related charges. Please contact oncore.support@yale.edu

8.. Are there any procedures involved in this protocol that will be performed at YNHH or one of its affiliated entities? Yes ___ No X *If Yes, please answer questions a through c and note instructions below. If No, proceed to Section III.*

a. Does your YNHH privilege delineation currently include the **specific procedure** that you will perform? N/A
b. Will you be using any new equipment or equipment that you have not used in the past for this procedure? N/A

c. Will a novel approach using existing equipment be applied? N/A

If you answered "no" to question 8a, or "yes" to question 8b or c, please contact the YNHH Department of Physician Services (688-2615) for prior approval before commencing with your research protocol.

Please note that if this protocol includes Yale-New Haven Hospital patients, including patients at the HRU, the Principal Investigator and any co-investigators who are physicians or mid-level practitioners (includes PAs, APRNs, psychologists and speech pathologists) who may have direct patient contact with patients on YNHH premises must have medical staff appointment and appropriate clinical privileges at YNHH. If you are uncertain whether the study personnel meet the criteria, please telephone the Physician Services Department at 203-688-2615. ***By signing this protocol as a PI, you attest that you and any co-investigator who may have patient contact has a medical staff appointment and appropriate clinical privileges at YNHH***.

SECTION III: FUNDING, RESEARCH TEAM AND TRAINING

1. **Funding Source:** Indicate all of the funding source(s) for this study. Check all boxes that apply. Provide information regarding the external funding source. This information should include identification of the agency/sponsor, the funding mechanism (grant or contract), and whether the award is pending or has been awarded. Provide the M/C# and Agency name (if grant-funded). If the funding source associated with a protocol is "pending" at the time of the protocol submission to the HIC (as is the case for most NIH submissions), the PI should note "Pending" in the appropriate section of the protocol application, provide the M/C# and Agency name (if grant-funded) and further note that University (departmental) funds support the research (until such time that an award is made).

PI	Title of Grant	Name of Funding Source	Funding	Funding Mechanism
David Fiellin, MD	Working with HIV clinics to adopt addiction treatments using Implementation Facilitation (WHAT IF?)	National Institute on Drug Abuse	☒ Federal<br>☐ State<br>☐ Non Profit<br>☐ Industry<br>☐ Other For Profit<br>☐ Other	☒Grant- R01 DA041067<br>☐Contract#<br>☐Contract Pending<br>☐ Investigator/Department Initiated<br>☐ Sponsor Initiated<br>☐ Other, Specify:

IRB Review fees are charged for projects funded by Industry or Other For-Profit Sponsors. Provide the Name and Address of the Sponsor Representative to whom the invoice should be sent. ***Note: the PI's home department will be billed if this information is not provided.***

Send IRB Review Fee Invoice To:

N/A

2. **Research Team:** List all members of the research team. Indicate under the affiliation column whether the investigators or study personnel are part of the Yale faculty or staff, or part of the faculty or staff from a collaborating institution, or are not formally affiliated with any institution. **ALL members of the research team MUST complete Human Subject Protection Training (HSPT) and Health Insurance Portability and Accountability Act (HIPAA) Training before they may be listed on the protocol. See NOTE below.**

NOTE: The HIC will remove from the protocol any personnel who have not completed required trainin

	**Name**	**Affiliation: Yale/Other Institution (Identify)**	**NetID**
**Principal Investigator**	David Fiellin, MD	Yale University	Daf7
**Role: Co-Investigator**	Lynn Fiellin, MD	Yale University	Les6
**Role: Co-Investigator**	E. Jennifer Edelman, MD	Yale University	Eje7
**Role: Co-Investigator**	Jeanette Tetrault, MD	Yale University	Tj46
**Role: Co-Investigator**	Philip Chan, MD	Brown University	N/A
**Role: Co-Investigator**	Deborah Cornman, PhD	University of Connecticut	N/A
**Role: Site PI**	Gabriel Rebick, MD	SUNY Downstate Medical Center	N/A
**Role: Consultant**	Peter Friedmann, MD	Baystate Medical Center	N/A
**Role: Biostatistician**	James Dziura, PhD	Yale University	Jdd7
**Role: Biostatistician**	Denise Esserman, PhD	Yale University	Dae6
**Role: Systems Analyst**	Laura Simone	Yale University	genovese
**Role: Biostatistician**	Tassos Kyriakides, PhD	Yale University	Drtk
**Role: Research Coordinator**	Evangelia Louizos	Yale University	el244

g. A personnel protocol amendment will need to be submitted when training is completed.

SECTION IV:
PRINCIPAL INVESTIGATOR/FACULTY ADVISOR/ DEPARTMENT CHAIR AGREEMENT

As the **principal investigator** of this research project, I certify that:

- The information provided in this application is complete and accurate.
- I assume full responsibility for the protection of human subjects and the proper conduct of the research.
- Subject safety will be of paramount concern, and every effort will be made to protect subjects' rights and welfare.
- The research will be performed according to ethical principles and in compliance with all federal, state and local laws, as well as institutional regulations and policies regarding the protection of human subjects.
- All members of the research team will be kept apprised of research goals.
- I will obtain approval for this research study and any subsequent revisions prior to my initiating the study or any change and I will obtain continuing approval of this study prior to the expiration date of any approval period.
- I will report to the HIC any serious injuries and/or other unanticipated problems involving risk to participants.
- I am in compliance with the requirements set by the University and qualify to serve as the principal investigator of this project or have acquired the appropriate approval from the Dean's Office or Office of the Provost, or the Human Subject Protection Administrator at Yale-New Haven Hospital, or have a faculty advisor.
- I will identify a qualified successor should I cease my role as principal investigator and facilitate a smooth transfer of investigator responsibilities.

As the **faculty advisor** of this research project, I certify that:

- The information provided in this application is complete and accurate.
- This project has scientific value and merit and that the student or trainee investigator has the necessary resources to complete the project and achieve the aims.
- I will train the student investigator in matters of appropriate research compliance, protection of human subjects and proper conduct of research.
- The research will be performed according to ethical principles and in compliance with all federal, state and local laws, as well as institutional regulations and policies regarding the protection of human subjects.
- The student investigator will obtain approval for this research study and any subsequent revisions prior to initiating the study or revision and will obtain continuing approval prior to the expiration of any approval period.
- The student investigator will report to the HIC any serious injuries and/or other unanticipated problems involving risk to participants.
- I am in compliance with the requirements set forth by the University and qualify to serve as the faculty advisor of this project.
- I assume all of the roles and responsibilities of a Principal Investigator even though the student may be called a PI.

____ ______________

Advisor Name (PRINT) and Signature Date

Department Chair's Assurance Statement

Do you know of any real or apparent institutional conflict of interest (e.g., Yale ownership of a

sponsoring company, patents, licensure) associated with this research project?

☐ Yes (provide a description of that interest in a separate letter addressed to the HIC.)

☐ No

As Chair, do you have any real or apparent protocol-specific conflict of interest between yourself and the sponsor of the research project, or its competitor or any interest in any intervention and/or method tested in the project that might compromise this research project?

☐ Yes (provide a description of that interest in a separate letter addressed to the HIC)

☐ No

I assure the HIC that the principal investigator and all members of the research team are qualified by education, training, licensure and/or experience to assume participation in the conduct of this research trial. I also assure that the principal investigator has departmental support and sufficient resources to conduct this trial appropriately.

______________________________________ ________________

Chair Name (PRINT) and Signature Date

____

Department

YNHH Human Subjects Protection Administrator Assurance Statement

Required when the study is conducted solely at YNHH by YNHH health care providers.

As Human Subject Protection Administrator (HSPA) for YNHH, I certify that:

- I have read a copy of the protocol and approve it being conducted at YNHH.
- I agree to notify the IRB if I am aware of any real or apparent institutional conflict of interest.
- The principal investigator of this study is qualified to serve as P.I. and has the support of the hospital for this research project.

____ ____________________

YNHH HSPA Name (PRINT) and Signature Date

SECTION V: RESEARCH PLAN

1. **Statement of Purpose:** State the scientific aim(s) of the study, or the hypotheses to be tested.

The specific aims with respect to increasing uptake of effective counseling and medications for tobacco, alcohol and opioid use disorders (addiction treatments) in four HIV clinics are:

Aim 1. Among key stakeholders, to use quantitative and qualitative (mixed) methods to identify the site-specific evidence, context and facilitation-related barriers and facilitators to the integration of addiction treatments to help tailor an Implementation Facilitation for each clinic.
Aim 2. To evaluate the impact of Implementation Facilitation on:

- 2a: Organizational readiness to deliver addiction treatments
- 2b: Provider readiness to deliver addiction treatments
- 2c: Provision of addiction treatments
- 2d: Changes in organizational models of care used to deliver addiction treatments

Aim 3. To evaluate the impact of Implementation Facilitation on ART receipt, viral suppression, VACS Index, and retention in HIV care among patients eligible for addiction treatment.

2. **Background:** Describe the background information that led to the plan for this project. Provide references to support the expectation of obtaining useful scientific data.

Why focus on tobacco, alcohol and opioid use disorders? These disorders are prevalent in HIV clinics, often co-occur,[54] and effective counseling and medication treatments exist.

HIV clinics rarely provide treatment for tobacco, alcohol and opioid use disorders
While effective addiction treatments exist, their use in HIV clinics is limited for a number of reasons at the patient, provider and organizational level. These include deficiencies in key stakeholder motivation, knowledge, prioritization and lack of a systematic approach to screening and treatment.[54,84,87-89]

Integrating addiction treatments into HIV clinics can improve substance use and HIV outcomes
Although there is limited literature on integrated treatment of HIV and substance use disorders in HIV clinics, evidence supports this approach.

Models for optimal integration of addiction treatment in HIV clinics may vary by organization and substance The implementation of addiction treatment into HIV clinics can be achieved using a range of models.[92] Using the concept of the medical home,[93] different models, using the skills of a range of HIV clinic providers such as nurses, social workers, physicians, nurse practitioners (NP), physician assistants (PA), pharmacists, and psychologists can support the integration of addiction treatment in HIV clinics.[94-96] The model that is optimally efficient and effective for each clinic may vary based on the substance, organizational, provider and patient-level factors. The significance of the Implementation Facilitation strategy (Defined below) we will evaluate is that it allows for the models to be developed based on a clinic level assessment of facilitators and barriers (formative evaluation) with input from relevant stakeholders, and provides flexibility such that models may be tailored depending on the substance being addressed, organizational factors, provider comfort, knowledge, skills and attitudes and patient input.

Implementation Science and the PARiHS framework can guide efforts to promote the uptake of addiction treatment in HIV Clinics

The field of Implementation Science can promote health care organization change and is gaining traction in the fields of HIV and Addiction Medicine.[97,98] Implementation Science, defined by the National Institute of Health as *the study of methods to promote the integration of research findings and evidence into healthcare policy and practice,*[99] provides organized approaches to help integrate addiction treatment into HIV clinics.[78,100] We will ground the development of our Implementation Facilitation in the Promoting Action on Research Implementation in Health Services (PARiHS) framework.[101] Our proposed research is consistent with a Hybrid Type 3 effectiveness-implementation study which focuses on simultaneously evaluating the impact of an implementation on provider and patient outomes.[30]

Implementation Facilitation leads to practice change

Defined as a "process of 'helping individuals and teams to understand what they need to change and how they need to change it in order to apply evidence to practice,'" Implementation Facilitation is an effective intervention[107] that includes a "deliberate process of interactive problem solving and support that occurs in the context of a recognized need for improvement and supportive interpersonal relationship."[49] (Table 2)

Research Plan: Summarize the study design and research procedures using non-technical language that can be readily understood by someone outside the discipline. **Be sure to distinguish between standard of care vs. research procedures when applicable, and include any flowcharts of visits specifying their individual times and lengths**. Describe the setting in which the research will take place.

The **W**orking with **H**IV clinics to adopt **A**ddiction **T**reatments using **I**mplementation **F**acilitation (**WHAT IF?**) study will evaluate the impact of Implementation Facilitation on the adoption of addiction treatment services in four HIV clinics. We will use a stepped wedge design (See Table 4). The stepped wedge design is a variation on a cluster randomized clinical trial in which participating sites are randomly assigned a time at which the intervention (Implementation Facilitation) is begun. The primary comparison is of the change that occurs from the pre-implementation period to two post-implementation periods, the initial six months (evaluation) and the following six months (maintenance). This proposal will consist of three main components in each of four clinics: 1) **formative evaluation** with key stakeholders at each site to guide and refine the Implementation Facilitation, 2) **conduct of the Implementation Facilitation** and 3) an **evaluation of the impact** of the Implementation Facilitation on organization and provider-level readiness, provision of addiction treatments, and HIV outcomes.

Intervention Sites

The sites are the Immunology Center at Miriam Hospital in Providence, RI, , Hartford Hospital's HIV Clinic in Hartford, CT, Haelen Center at Yale-New Haven Hospital, SUNY Downstate Medical Center' HIV clinic in Brooklyn, NY. All sites have conducted research using their electronic medical records.[144,145,176,177] The diverse sites' commonalities and differences will drive local modifications to the Implementation Facilitation and enrich its generalizability. Table 3 provides estimates, where known, of the current need for and provision of addiction treatments

at the four sites. Currently, approximately 15% of eligible patients are receiving addiction treatments.

Table 3. Site Descriptions, Current Need and Provision of Addiction Treatments

Site	Miriam Immunology Center	Boston Medical Center	Hartford Hospital	Downstate
**Number of HIV-infected receiving care**	1600	1200	500	1200
**Number with untreated Tobacco Use Disorder**	400	300	200	425
**Number receiving on-site medication for tobacco use disorder (bupropion, NRT, varenicline)**	140	Unknown	75	110
**Number receiving on-site counseling for tobacco use disorder**	Unknown	Unknown	50	7
**Number with untreated Unhealthy Alcohol Use**	100	200	75	240
**Number receiving on-site medication for unhealthy alcohol use (naltrexone, acamprosate, disulfiram)**	0	16	0	0
**Number receiving on-site counseling for unhealthy alcohol use**	Unknown	16	0	100
**Number with untreated Opioid Use Disorder**	80	100	80	100
**Number receiving on-site medication for opioid use disorder (buprenorphine, naltrexone)**	32	50	0	4
**Number receiving on-site counseling for opioid use disorder**	32	50	0	7

Formative Evaluation (Aim 1)

Overview of Formative Evaluation

We will conduct a three-stage formative evaluation using mixed-methods[178] (qualitative and quantitative) to identify evidence, context, and facilitation-related factors impacting the provision of addiction treatments and use these data to tailor, refine, monitor and evaluate the effectiveness of the Implementation Facilitation. Given that formative evaluation techniques will be used in advance, during and following Implementation Facilitation, we will describe the methods in detail here and refer back to this section as needed.

Formative evaluation is a widely accepted implementation assessment approach designed to identify influences on the development, progress and effectiveness of implementation efforts.[109] To increase use of addiction treatments, we will use this formative evaluation to understand: 1) site-specific practices, 2) determinants of these practices, 3) barriers and facilitators to practice change, and 4) perspectives regarding Implementation Facilitation. Using the PARiHS framework, we will employ a mixed-methods approach with 1) quantitative methods, using the Organizational Readiness to Change Assessment (ORCA)[105] followed by 2) qualitative methods to develop an understanding of evidence, context and facilitation-related factors impacting the provision of addiction treatments from the perspectives of diverse stakeholders including patients, nurses, social workers, physicians, NPs, PAs, pharmacists, psychologists, clinic administrators, payers and clinic directors.

Eligibility for Participation in Formative Evaluation:

Patients: HIV-infected; age $\geq$18 years old; meets criteria for lifetime or current tobacco, and/or alcohol and/or opioid use disorder regardless of addiction treatment status; able to provide verbal informed consent.

Clinic Staff, Administrators: employed at participating HIV clinic for at least six months.
Payers: employed at an organization or agency that provides funding for medical services for HIV-infected individuals for at least 6 months.

Quantitative Data Collection and Analysis

We will use the ORCA,[105] a 77-item instrument based on the PARiHS framework, to evaluate evidence, context and facilitation-related factors impacting implementation of addiction treatments in each of the four HIV clinics. Baseline ORCA scores will be used to determine evidence, context and facilitation-related strengths and weaknesses in organizational readiness to implement addiction treatment and to tailor the Implementation Facilitation.

Qualitative Data Collection and Analysis

We will conduct focus groups with a purposeful sample of key stakeholders at three distinct stages of our project: the planning phase, implementation phase and evaluation phase. Purposeful sampling is a well-established method in qualitative studies and is designed to identify study participants who have direct experience with or knowledge of the phenomenon of interest, in this case substance use disorders and addiction treatment. We will ensure appropriate breadth of samples with regard to salient characteristics that may influence participant views on the topics of interest. We have chosen to use focus groups given their suitability for generating data from multiple perspectives regarding the organizational and individual level factors impacting complex processes whereby the group interaction is anticipated to stimulate unique ideas.[178] We will enroll patients, nurses, social workers, physicians, NPs, PAs, pharmacists, psychologists, clinic administrators, payers and clinic directors at each site to allow for evaluation of processes from multiple perspectives (triangulation). Focus groups will be conducted at each site with 8-10 patients with past or current tobacco, alcohol and/or opioid use disorders and 8-10 staff members and payers, with at least one individual from each of the stakeholder categories. We will conduct focus groups until we reach thematic saturation.[182]

Development of focus group questions: The focus group guides will be informed by the PARiHS framework and include "grand tour" questions designed to establish rapport and elicit open-ended responses. Probes will be used to understand specific details of those experiences and allow for clarification of ideas. We will design and pilot test these guides with patients and key informants from the New England HIV Implementation Science Network and refine as indicated.

Conduct of focus groups: Dr. Edelman, in conjunction with a trained research assistant, will conduct focus groups at each site during year 1 (planning phase), year 2-3 (implementation phase) and year 4 (evaluation and maintenance phases). The focus groups will be recorded and professionally transcribed for qualitative analysis. Participants will also complete a brief anonymous demographic survey. Using directed content analysis,[163] we will analyze the data with a multi-disciplinary group with experience in qualitative methods, which will include HIV

and Addiction Medicine physicians, a HIV Clinic Director, a Clinical Psychologist, and a health service researcher. Data will be entered into and organized using Atlas.ti software.

We will share our results with participants (participant confirmation) to ensure that we have accurately understood and represented stakeholder's perspectives and experiences. We will use data reduction strategies to sharpen, sort, focus, discard and organize data in a way to draw inferences regarding the implementation interventions in place at each facility. We will develop a template summary of data at each facility organized by evidence, context and facilitation-related factors. Once we have developed that summary for each site, we will create a matrix across all sites and respondents to understand the major issues with regard to implementation across sites. These data will directly inform the site-specific Implementation Facilitation and be used to evaluate the facilitation process and outcomes.

Description of the implementation strategy

Overview of the Implementation Facilitation (See Table 2)

Implementation Facilitation will be based on a manualized program developed by Kirchner and colleagues[106] that has had significant impact on implementing healthcare practices in clinical settings. Building on the mixed-methods analysis conducted in Aim 1, we will use the PARiHS framework to tailor the Implementation Facilitation for site-specific needs. The facilitators and barriers identified by administrators, providers, and patients and will be characterized according to the PARiHS sub-elements of patient and clinical experience (communication, knowledgeable and empathetic providers), receptive context (resources to provide addiction treatments), and culture (value of team-based approach) identified. As described below, PARiHS will be used to further explicate and design the Implementation Facilitation, guide the ongoing formative evaluation and revise the strategy in an iterative manner to improve implementation success. The individual components (Table 2) of Implementation Facilitation are described below.

Table 2. Components of Implementation Facilitation

Component	Description
**External Facilitator**	Outside content expert who assists site
**Local Champion**	Local site stakeholder who promotes change
**Provider Education and Academic Detailing**	Provision of unbiased peer education
**Stakeholder Engagement**	Aligning goals of implementation and those impacted
**Tailor Program to Site**	Addressing site specific needs based on Aim 1
**Performance Monitoring and Feedback**	Assess implementation of screening and treatment efforts and inform site of results
**Formative Evaluation**	Quantitative and qualitative determination of impact
**Establish a Learning Collaborative**	Shared learning opportunities tailored to stakeholders
**Program Marketing**	Efforts designed to increase attention to availability of on-site addiction treatment services

Components of Implementation Facilitation

External facilitator - Dr. Edelman will provide training, coach and mentor local champions, and encourage the exchange of ideas within and among sites.

Local champions - Dr. Edelman will spend the first year of the project working with the local site PIs to identify and engage local champions, including physicians, clinic directors, pharmacy or nursing leads or local organizational re-design/quality improvement experts. These

individuals, and their current level of involvement in the HIV clinic, will likely vary by site and substance. Following the in-person orientation and trainings, Dr. Edelman will provide external facilitation including monthly phone meetings for the six months of the Implementation Facilitation. The Project Coordinator will take notes during these meetings, capturing information on challenges, barriers, facilitators and strategies. This information will be integrated into the formative evaluation.

Provider education and academic detailing - Academic detailing involves trained clinician consultants visiting other clinicians to share unbiased information about patient assessment and treatment with the goal of improving quality of care.[106] All providers involved in the implementation will have dedicated educational sessions on addiction treatments specifically tailored to each provider's tasks based on the initial formative evaluation and potentially modify, remove or add strategies to enhance implementation. We will address practical issues such as efficient use of the electronic medical record, public, private and AIDS Drug Assistance Program payment for treatments, and patient monitoring strategies. We will share protocols for integration that have been developed for integration of buprenorphine and naltrexone at the Yale HIV clinics and elsewhere. Drs. D. Fiellin, L. Fiellin, Tetrault, and Edelman will be primarily responsible for providing the content or identifying local site content experts who can deliver interactive training sessions. Training strategies will be based on adult learning theory and include didactic presentations on the effectiveness and safety of addiction treatment and skill's based practice sessions. These sessions will focus on 1:1 and small group activities to promote the education of providers regarding the use of motivational interviewing, brief interventions, addiction counseling and medications. We will focus on frequent brief presentations at provider meetings, lectures with meals/refreshments and provide instructional handouts.

Stakeholder engagement will take place at the administrative, provider, and patient level. Efforts at increasing engagement will be informed by the focus groups conducted during the initial formative evaluation and supported by the efforts of the local champions. This work will be informed by the Normalization Process Model[183] whereby the external facilitators will work to make attention to tobacco, alcohol and opioid use disorders a routine focus of stakeholders (e.g. embedded) and processes are developed whereby practices such as routine screening and medication use are sustained and routinely monitored.

Tailoring the program to local site will occur as a result of the formative evaluations and will be informed by local site PIs and local champions.

Performance monitoring and feedback - This will involve regular assessment of individual clinician performance and providing information about that performance.[106] We will work with clinic directors and other members of the clinic team to identify the optimal outcomes to be tracked as well as how often and in what format feedback will be provided based on clinic and provider-level data. Outcomes will include the number of screenings performed, prescriptions of addiction medications provided or number of sessions of the counseling provided. Dr. Edelman in collaboration with Drs. D. and L. Fiellin and Dr. Tetrault will provide additional training or educational booster sessions for sites with low implementation and those requesting such services.

Implementation-focused formative evaluation will occur during the implementation and focus on the discrepancies between the implementation plan and its operationalization. Examples of processes that will be examined include: 1) number and types of educational trainings attended by staff, 2) number of staff who view the audit and feedback reports, and 3) number of champions that attend facilitation meetings.

Progress-focused formative evaluation meetings**,** led by Dr. Edelman, will monitor achievement of implementation goals and performance targets to identify blocked progress, allowing steps to be taken to optimize the intervention. These meetings will focus on barriers and strategies to address identified barriers. Non-attendance of site participants will be documented and outreach through individual facilitation meetings, calls or emails will be initiated to assess for stalled progress and offer assistance.

Interpretive formative evaluation uses the data collected from the other formative evaluations and information collected at the end of the project regarding the participant experiences to clarify the meaning of successful or failed implementation and to enhance understanding of Implementation Facilitation's impact. At the conclusion of the maintenance phase (12 months), we will conduct an interpretive evaluation that will assess stakeholder views regarding (a) value of each addiction treatment, (b) satisfaction or dissatisfaction with various aspects of Implementation Facilitation, (c) reasons for clinic level action or inaction, (d) additional barriers and facilitators, and (e) recommendations for further refinements. Information will also assess stakeholders' beliefs regarding Implementation Facilitation's success and overall ''worth''.[109]

A Learning Collaborative will be formed by inviting each of the sites' local champions, and other key stakeholders, to participate in a monthly call to promote shared learning regarding issues promoting and hindering implementation of addiction treatment. The local champions and key stakeholders will set the agenda and the calls will be facilitated by Dr. Edelman and Dr. Fiellin and provide a dedicated time to discuss site-specific updates, challenges and possible solutions for implementation of addiction services. Dr. Edelman will make resources, protocol templates, materials and presentations available through the New England HIV Implementation Science Network and other websites so they will be widely accessible.

Program Marketing will be conducted to promote awareness among patients and providers in the clinics of the availability of on-site addiction treatment services. This will include dissemination of promotional materials such as "What if? Ask me!" buttons, flyers, pens, pads and sticky notes for all clinic staff (Appendix for examples from current studies). The goal of the marketing will be to promote screening and treatment. We will emphasize patient motivation in this process. While the efficacy of addiction treatment is clear, most research is conducted on motivated patients seeking treatment for their addictive disorders. Our experience conducting research in HIV clinics and general medical settings demonstrates that patient motivation to address their addictive disorder is often very low, especially when their disorder is detected during a routine visit for a general medical condition.[18,37-39,41] This means that it will be imperative for our efforts to increase treatment of addictive disorders in HIV settings to address patient motivation. Our "What if? Ask me!" slogan is designed to initiate a motivational discussion between patients and providers. The ultimate marketing approach will be tailored to the needs of the stakeholders and include direct (e.g. "in-services" to discuss the treatment

services and referral processes, flyers) and indirect (e.g. informal conversation) approaches. We will distribute a newsletter every six months to provide updates about the “WHAT IF?” program across the sites.

Please note that these materials will not be designed until April 2016. At that time, an amendment will be submitted to the IRB for approval.

Evaluating the Effectiveness of the Implementation Facilitation (Aim 2 and Aim 3)
Overview
We will use a stepped wedge design (Table 4) to evaluate the effect of the Implementation Facilitation on the outcomes. In a stepped wedge design, clinics are randomly assigned to time cohorts and then followed prospectively to determine their outcome status. Stepped wedge designs are increasingly used to promote the implementation of evidence-based practice and are appropriate for evaluating interventions where there is evidence that the intervention is likely to be beneficial and when practical considerations require sequential deployment of an intervention.[50,184] We will have three phases of six-months each: 1) a pre-implementation control period, 2) a post-intervention evaluation period, and 3) a maintenance period. The proportion of eligible patients in each time period who receive at least one addiction treatment will be assessed and the effect of the intervention will be evaluated by comparing pre-implementation to evaluation and maintenance periods. This will allow us to assess the short and long-term (maintenance) impact of Implementation Facilitation.

Table 4. Overview of clinic phases the stepped wedge study design (IF = Implementation Facilitation)

**Clinic 1**	Control	Control	Control	Control	IF	Evaluation	Maintenance
**Clinic 2**	Control	Control	Control	IF	Evaluation	Maintenance	Maintenance
**Clinic 3**	Control	Control	IF	Evaluation	Maintenance	Maintenance	Maintenance
**Clinic 4**	Control	IF	Evaluation	Maintenance	Maintenance	Maintenance	Maintenance

We will measure a range of implementation variables at baseline, 6 and 12 months (Table 5). Using validated instruments before, during and after Implementation Facilitation we will examine the impact of Implementation Facilitation on organization readiness, provider readiness, treatment provision, models of care and HIV outcomes. For sample size calculation, the primary quantitative outcome, assessed six and 12 months after the Implementation Facilitation is completed, will be the proportion of eligible individuals receiving addiction treatments from the clinic measured using information derived from the electronic medical record. This determination will be conducted in a blinded fashion, such that the individual analyzing the electronic medical record data will be unaware of the phase (control, evaluation, maintenance) of the Implementation Facilitation.[50] Secondary quantitative outcomes, assessed at six and 12 months will be change in organizational readiness measured using the ORCA, change in provider readiness to prescribe addiction medications measured using change rulers, and HIV-related outcomes. To facilitate data collection, all surveys will be administered to all stakeholders online using the survey software, Qualtrics and collected anonymously.

Table 5. Summary of Study Assessments

Assessment	Data Source	Baseline Control	6 months Evaluation	12 months Maintenance
**Organizational Readiness to Change Assessment**	Provider and Organizational representatives	X	X	X
**Change Rulers for each medication and counseling intervention**	Provider and Organizational representatives	X	X	X
**Conduct of screening for each condition**	Electronic Medical Record	X	X	X
**Use of each medication and counseling intervention**	Electronic Medical Record	X	X	X
**Patient demographic information**	Electronic Medical Record	X	X	X
**Receipt of antiretroviral therapy**	Electronic Medical Record	X	X	X
**Tobacco, Alcohol and Opioid Use Disorders**	Electronic Medical Record	X	X	X
**Psychiatric Diagnoses**	Electronic Medical Record	X	X	X
**Clinic Visits**	Electronic Medical Record	X	X	X
**Laboratory values**	Electronic Medical Record	X	X	X
**Receipt of benzodiazepines**	Electronic Medical Record			X
**Receipt of COVID-19 screening and COVID-19 diagnosis**	Electronic Medical Record			X

Adequacy of sample size

Based on prior work,[78,79,106,107] we anticipate that Implementation Facilitation will increase the proportion of eligible patients provided addiction treatment by the clinics from a baseline of 15% of eligible patients (Table 3) to 26% during the evaluation phase and 33% during the maintenance phase. This reflects an 11% absolute increase (75% relative increase) during the evaluation phase and a 19% absolute increase (125% relative increase) during the maintenance phase. A parallel group design, unadjusted for clustering and repeated measures would require a sample size of 592 (296 in each the control and intervention arms) to detect the estimated effect size (15% control vs. 26% intervention; power 90% and Type I error (alpha) of 0.05). We used the formula of Woertman et al. to incorporate the effect of the stepped wedge design.[184] It was assumed that each clinic would provide a minimum of 300 addiction treatment eligible patients (n). In addition, the following assumptions were used, intracluster correlation, $\rho = 0.01$, number of steps, $k = 4$, number of baseline measurements, $b=1$, number of measurements taken after each

step, t=1. With the above assumptions, the derived DE_{SW} = 0.63, thus yielding an adjusted sample size of 375 across the 4 clinics. This yields a sample size for the initial evaluation phase of 94 patients per clinic who are eligible for addiction treatment (tobacco, alcohol or opioid use disorder). Three primary time points will be used, control (six months) evaluation (six months) and maintenance (six months). Given we will have adequate power to detect a difference of a 11% increase in the provision of addiction treatments in the evaluation phase, we will be adequately powered for the 19% increase anticipated during the maintenance phase and for the other quantitative analyses proposed in Aims 2 and 3. This sample size estimated above is based on a ‘cross-sectional’ design. Even if the population in the clinics/clusters is assumed to be a stable ‘cohort’, the power will likely be more than sufficient due to within-patient correlation.

Pre-implementation and serial assessments

Organizational Readiness: We will use the ORCA to measure factors impacting the provision of addiction treatments. This 15-minute survey is based on the PARiHS framework and asks the respondent to rate local factors related to evidence, context and facilitation on a 5-point Likert scale from strongly disagree to strongly agree. Facilitation questions will be omitted from the pre-implementation assessment since this part of the intervention and will not have taken place.

Provider Readiness: We will use change rulers, among appropriate providers, for each of the medications and counseling to assess readiness to provide each medication and counseling intervention.[185] The change rulers will independently assess, on a 0-10 scale each provider’s: 1) confidence to prescribe/provide the intervention, 2) readiness to prescribe/provide the intervention and 3) commitment to prescribe/provide the intervention. Stage of change assessments have been validated and have been used in the field of addiction and mental health to assess readiness to adopt evidence-based treatments.[186-189]

Addiction Treatment Eligibility: We will work with each site to incorporate screening for smoking and use of the AUDIT-C,[190] NIAAA single question screen or NIDA-MED ASISST[191]. The NIDA-MED ASISST and NIAAA screen have good operating characteristics for both alcohol and drug use disorders in medical settings.[192-194] We will use electronic medical records to identify addiction treatment eligible patients based on ICD-9 diagnoses for current (or in remission, at risk for relapse) substance use disorders, positive NIDA-MED ASISST or for tobacco - smoking 5 or more cigarettes/day and alcohol - AUDIT-C > 3, positive NIAAA screen.

Provision of Addiction Treatments: We will use the electronic medical record to identify prescriptions for each of the target medications in the surveillance period. We will use CPT codes to determine whether specific counseling therapies were provided.

3. **Genetic Testing N/A** ☒
   - **A.** Describe
     - i. the types of future research to be conducted using the materials, specifying if immortalization of cell lines, whole exome or genome sequencing, genome wide association studies, or animal studies are planned
     - ii. the plan for the collection of material or the conditions under which material will be received

iii. the types of information about the donor/individual contributors that will be entered into a database
iv. the methods to uphold confidentiality

B. What are the conditions or procedures for sharing of materials and/or distributing for future research projects?
C. Is widespread sharing of materials planned?
D. When and under what conditions will materials be stripped of all identifiers?
E. Can donor-subjects withdraw their materials at any time, and/or withdraw the identifiers that connect them to their materials?
    i. How will requests to withdraw materials be handled (e.g., material no longer identified: that is, anonymized) or material destroyed)?

F. Describe the provisions for protection of participant privacy
G. Describe the methods for the security of storage and sharing of materials

4. **Subject Population:** Provide a detailed description of the types of human subjects who will be recruited into this study.

Participants for patient focus groups will be an estimated 24-30 HIV-infected men and women with a history of tobacco, alcohol or opioid use disorder who receive care in one of the three HIV clinics. These individuals will participate in focus groups to determine their perspective on the optimal ways to integrate treatment for tobacco, alcohol and opioid use disorders into HIV clinics. We will also enroll an estimated 240 staff (clinicians and administrators) across the four sites to participate in focus groups and complete on-line quantitative assessments.

5. **Subject classification:** Check off all classifications of subjects that will be <u>specifically recruited for enrollment</u> in the research project. Will subjects who may require additional safeguards or other considerations be enrolled in the study? If so, identify the population of subjects requiring special safeguards and provide a justification for their involvement.

☐ Children ☐ Healthy ☐ Fetal material, placenta, or dead fetus
☐ Non-English Speaking ☐ Prisoners ☐ Economically disadvantaged persons
☐ Decisionally Impaired ☐ Employees ☐ Pregnant women and/or fetuses
☐ Yale Students ☐ Females of childbearing potential

NOTE: Is this research proposal designed to enroll children who are wards of the state as potential subjects? ☐ Yes ☒ No (If yes, see Instructions section VII #4 for further requirements)

6. **Inclusion/Exclusion Criteria:** What are the criteria used to determine subject inclusion or exclusion?

Patient inclusion criteria:

1. HIV-infected
2. Receiving HIV care in the index clinic
3. Age ≥18 years old
4. Meets criteria for lifetime or current tobacco, alcohol and/or opioid use disorder regardless of addiction treatment status
5. Able to provide verbal informed consent

Staff inclusion criteria:
1. Employed at participating HIV clinic for at least 6 months
2. Able to provide verbal informed consent.

Payer inclusion criteria:
1. Employed at an organization or agency that provides funding for medical services for HIV-infected individuals for at least 6 months.
2. Able to provide verbal informed consent.

Exclusion criteria: Unable to provide verbal informed consent

7. How will **eligibility** be determined, and by whom?

Patients, Staff and Payers:
Local Research Assistants will recruit patients, staff and payers from their HIV clinical care sites and determine eligibility.

8. **Risks:** Describe the reasonably foreseeable risks, including risks to subject privacy, discomforts, or inconveniences associated with subjects participating in the research.

Risk to subjects is minimal as the intervention proposed is consistent with quality improvement efforts and all analyses will be performed with attention to privacy and security. The participants in the focus groups will be provided with an information sheet outlining the purpose of the study and their ability to opt out at any time. The Site PI or Research Assistant will obtain informed consent from site staff participating in the focus group interviews. The rating scales and questionnaires are all non-invasive, and should also add no risks to subjects, as our past experience indicates.

9. **Minimizing Risks:** Describe the manner in which the above-mentioned risks will be minimized.

Numerous steps will be taken to protect confidentiality. In addition, a Certificate of Confidentiality has been obtained from NIDA to protect information collected from patients during the conduct of the focus groups. This certificate will protect the confidentiality of all patient level data by this study.

Recruitment and informed consent:
There are three aspects to the study:

I. Focus Groups with patients, clinic staff and payers
II. Online survey based on the measure ORCA – clinic leadership staff and providers
III. Patient Outcomes: Evaluation of intervention on patient level outcomes using patient-level data from the electronic medical records

I. Focus Groups
Local Research Assistants will recruit patients from their HIV clinical care sites to participate in focus groups. If they are interested, participants will be asked to come to a private office where they will meet with a Research Assistant who will describe the study in more detail, determine interest in participating, and assess potential eligibility. After fully informing participants about the study and answering any questions, the Research Assistant will obtain verbal informed consent.

There may be a perceived risk among HIV clinic staff that participation in this research may impact their employment status in a negative way. To address this concern, we will work with the HIV clinics to provide staff assurances that their participation in the research will in no way affect their employment status either positively or negatively. Surveys and focus groups will be voluntary, and participants will be free to skip any questions they do not wish to answer. Information collected for research purposes will not become part of staff's personnel records. The informed consent for the HIV clinic staff form will outline these assurances. For the recording of the focus group sessions, participants will be made aware during the informed consent process they will be digitally recorded and, for patients, that the nature of these sessions will involve participants speaking about information regarding their health status, smoking, alcohol use, and opioid use, HIV status, and medical history.

Audio recordings will be stored on a password protected computer. The research team and the professional transcriptionists will use the Yale Secure Transfer File website to send recordings and transcripts. Identifying information from any of the participants in the formative evaluations will be kept separately from any forms on which they record information. References to identifying information will be eliminated from the written transcript of the interview in preparation for analysis of qualitative data. Names and any other identifying information collected on evaluation forms, meeting minutes, or field notes and document reviews will be eliminated in preparation for analysis of these records. These precautions are expected to be completely effective in eliminating risks to confidentiality.

Recorded sessions: Participants will be made aware during the informed consent process that focus groups will be digitally recorded. However, participants will be instructed to avoid using names and the recordings will be coded by randomly generated group numbers to protect participant confidentiality. Participants will be given the option to have their information redacted from transcripts. The transcripts will be reviewed by Dr. Edelman or the Research Coordinator and all personally identifying information will be removed prior to analysis. All appropriate actions will be taken by staff members to minimize the risks associated with loss of confidentiality. Audio files will be coded by number and will be erased.

II. Online Survey
Survey data: Information obtained from sites will include descriptive information and research assessments. Access to this information will be limited to study staff. Analytic datasets will be created with study identification codes. There will be an online consent for participants.

III. Patient Outcomes
Electronic medical record data: We will only collect aggregate and de-identified data from the electronic medical record. Any potentially individual identifiable health information will be protected in accordance with the Health Insurance Portability and Accountability Act (HIPAA) of 1996. All research personnel will be trained on Institutional Review Board (IRB) and HIPAA procedures.

In Case of Injury: If a participant is injured as a direct result of participation in this study, treatment will be provided. The participant and/or his or her insurance carrier will be expected to pay the costs of this treatment. No additional financial compensation for injury or lost wages is available. Participants will not waive their legal rights by participating in this study.

10. **Data and Safety Monitoring Plan:** Include an appropriate Data and Safety Monitoring Plan (DSMP) based on the investigator's risk assessment stated below. (Note: the HIC will make the final determination of the risk to subjects.) For more information, see the Instructions, page 24.
    a. What is the investigator's assessment of the overall risk level for subjects participating in this study? **Minimal**
    b. If children are involved, what is the investigator's assessment of the overall risk level for the children participating in this study? N/A
    c. Include an appropriate Data and Safety Monitoring Plan. Examples of DSMPs are available here http://www.yale.edu/hrpp/forms-templates/biomedical.html for
        i. Minimal risk
        ii. Greater than minimal

Data and Safety Monitoring Plan:
The risk of loss of privacy is minimal. Measures in place to prevent the loss of privacy include password protection on data files and computer workstations.
Below we detail the data center architecture, backup strategies, and physical data storage that will support the protection of our data.

Data Monitoring and Storage:
We are planning to use a Web-based computer system for data collection, monitoring and reporting. This system has proven to be very efficient and reliable in clinical trials being conducted by our research team. The core of this system consists of a database hosted on a network of secure servers that are capable of collecting and storing data using Web based applications that are accessed by the remote users via standard Internet browsers. The Web based

data system is designed to fulfill strict requirements of a clinical trial regarding data collection, monitoring, and reporting from the recruitment of participants to the delivery of data sets suitable for statistical analyses at the end of the trial. The system meets the highest security and reliability standards. All connections to the systems are secured and encrypted and only authorized users are able to access the system. All data is stored in encrypted files, and multiple backups of the system and all data are maintained.

Data and safety monitoring procedures in this study include computerized data collection and monitoring systems and an organizational structure of clearly defined tasks assigned to all research and clinical personnel involved in the conduct of this study. The computerized data collection and monitoring system consists of a database system that records research activities, completion of scheduled assessments, and delivers computerized versions of most of research instruments used in this study. Research assistants use this database to monitor and schedule activities and to administer study assessments. Data entry of non-computerized assessments is accomplished by using specialized data entry software (such as, SPSS Data Entry or Microsoft Access Data base) facilitating efficient data entry and allowing elimination of out-of range values and double entry of data for detection of key punch errors.

The organizational structure used to ensure quality of data in this project include: 1) extensive training and close supervision of research assistants in data collection; 2) preliminary review of all data for completeness and coding errors by data manager/analyst; and 3) utilization of error-checking statistical procedures.

Experienced data manager/analysts and the PI supervise data procedures. The research team will meet weekly to review the overall progress of the study. All error corrections are fully documented in the research records of the study. All research personnel are required to participate in and document training in protection of human subjects and the responsible conduct of scientific research. Procedures for training and supervision of Research Assistants, and transcriptionists have been developed as part of our previous and current studies. Initial training utilizes intensive seminars on all of the research instruments, after which the coordinator observes the trainer administer the assessments and co-rates the assessments. Subsequently, the coordinator will conduct the assessments and make the ratings with the trainer present for a minimum of five full assessments and until complete agreement is obtained. During the study, the Yale-based Research Coordinator will review all survey assessments and provide feedback on the completeness, accuracy or errors forms. Continued supervision and training on all instruments is provided on a regular basis to insure continued reliability of the assessments.

d. For multi-site studies for which the Yale PI serves as the lead investigator:
   i. How will adverse events and unanticipated problems involving risks to subjects or others be reported, reviewed and managed?

All adverse events are reported using the Yale Institutional Review Board (IRB) standard template for reporting adverse events. The Yale Principal Investigator (PI) reviews all adverse events, classifies the attribution of adverse events (e.g., definitely, probably, possibly related; unlikely or unrelated) and grades the severity of the event, utilizing the FDA's definition of serious adverse events, on a 6-point scale (0=no adverse event or within normal limit; 1=mild; 2=moderate; 3=severe; 4=life-threatening; 5=fatal). Serious, definitely protocol related adverse events will be

reported immediately to the IRB and to NIDA. Adverse events will be reported in summary form at least annually to the IRB. The summary will include the number of subjects enrolled and a summary of graded adverse events to date, using the chart format included in the Yale University DSMP template. The PI will evaluate all adverse events and determine whether the event affects the Risk/Benefit ratio of the study and whether modifications to the protocol (e.g., Risks to Subjects) or consent form (e.g., Risks and Inconveniences) are required.

ii. What provisions are in place for management of interim results?

No interim analyses will be performed.

iii. What will the multi-site process be for protocol modifications?

There will be one protocol document and each participating institution will utilize that document. The Yale PI is responsible for the coordination of the approval of the protocol as well as its subsequent amendments and will be responsible to ensure that the sites are using the correct version of the protocol. The Yale PI will collect and maintain copies of all IRB approvals from each site and will collect and review conflicts of interest declarations made by the Site PIs.

11. **Statistical Considerations:** Describe the statistical analyses that support the study design.

General considerations: This is a stepped wedge randomized trial to evaluate the impact of Implementation Facilitation. Randomization and data analysis will be conducted by Drs. Dziura, Kyriakides and the Yale Center for Analytic Sciences (YCAS). All analyses will be carried out using SAS v9.3 (SAS Institute, Cary, NC) and an alpha level of 0.05 will be used to establish statistical significance.

Interim monitoring: Interim monitoring will focus on adherence to protocol, completeness of data retrieval from each clinics electronic medical record, and uptake of the Implementation Facilitation. A set of monitoring tables will be generated by YCAS for this purpose. No interim looks for efficacy are planned.

Analysis for Aim 2a, 2b, and 2c: Number (percent) will be used to present data from categorical variables. Continuous variable data will be presented as means (+/- SD) and those with a non-normal distribution will be characterized using medians (interquartile range). Differences across the clinics will be assessed using appropriate parametric or non-parametric techniques. The provision of addiction treatments among those eligible (Aim 2c) will be expressed as a percentage and the distribution of results in the control (unexposed) periods will be compared with those in the evaluation (exposed) and maintenance (exposed) periods. Characteristics of patients and clinics will be shown by randomization status in each step of the design. We will conduct similar analyses for the measures of organizational and provider readiness (Aims 2a, 2b) where the dependent variable will be expressed as continuous measures (ORCA score and change rulers). Based on the PARiHS, we hypothesize that stakeholder scores on the ORCA subscales including clinical experiences, patient preferences, leadership culture

and measurement will mediate the proportion of patients receiving addiction treatment. We will test the relationship among this outcome and these mediators. To test the significance of the mediation effect, which is called the indirect effect, we will calculate Sobel's test for each proposed mediator. We will use an intent-to-treat approach (i.e. cluster analysis will be done) according to the time clinics were intended to cross over from control to Implementation Facilitation. A linear mixed model will be used, adjusting for calendar time (a potential confounder due to its association with both exposure to the intervention and outcome), with a random effect for clinic, a fixed effect for each step and allowing for repeated measures for patients in the clinics. Since the population of the clinics participating in the proposed trial will be rather stable, thus rendering this a cohort stepped wedge design, an additional random effect for patients in each clinic will be introduced.

Analysis for Aim 2d: To determine the impact of Implementation Facilitation on models of care we will describe the extent to which the provision of treatment for each substance is coordinated (facilitated by the HIV clinic), co-located (provided in the clinic) or integrated (provided by the primary HIV provider) by clinic. We will track changes in these models of care from the control period to the evaluation and maintenance periods.

Analysis for Aim 3: These analyses will be restricted to patients diagnosed with a tobacco, alcohol or opioid use disorder. ART receipt will be defined as at least 180 days of three concurrent antiretroviral agents in the 6-month interval based on prescriptions in the electronic medical record. Viral suppression, will be defined as HIV RNA <200 copies/mL at last test closest to the time of data extraction, consistent with Department of Health and Human Services (DHHS) guidelines. Although current limits of detection are lower, this cutoff allows for low-level "blips" that have no clinical significance. The VACS Index will be evaluated based on most recent values at the time of data extraction. VACS Index score will be treated as a continuous variable. Retention in HIV care, per DHHS guidelines uses a 24-month measurement period. It is defined as at least one HIV medical care visit in each 6-month period, with a minimum of 60 days between the first medical visit in the prior 6-month period and the last medical visit in the subsequent 6-month period. This measure has good agreement with the Institute of Medicine indicator for retention in care and is an independent predictor of mortality. We will evaluate whether patients have had at least 1 visit in the 6-month period prior to time of data extraction. These analyses will be adjusted for demographics (age, gender), and presence of psychiatric diagnosis (anxiety, major depression, severe mental illness). We will use descriptive statistics to characterize addiction treatment eligible patients overall and by substance at baseline and examine changes at each interval. We will then examine each of these HIV-related outcomes at the 6-month intervals of control, evaluation and maintenance phases based on the provision of addiction treatment. We will determine the association between provision of addiction treatment, including benzodiazepines and outcomes with the MIXED models procedure repeated measures and generalized estimating equations (GEE) to account for repeated measures for dichotomous outcomes. We will adjust for fixed demographics, substance use disorder and psychiatric diagnoses. We will describe the association with odds ratios and 95% confidence intervals. For continuous outcomes, we will use multivariable linear regression with GEE with similar adjustment.

Plan for Missing Data: Several strategies will be imposed to accommodate the likelihood that missing data will occur during this study. Prevention is the most obvious and effective manner to control bias and loss of power from missing data. We will follow the intent to treat principle, requiring follow-up of all clinics randomized regardless of the treatment received. Timely data entry combined with quarterly missing data reports will trigger protocols for tracking and obtaining missing data. Despite these efforts it is reasonable to assume missing data will occur. Our primary analysis is valid under the assumption that missing data is missing at random (MAR). We will evaluate the plausibility of this assumption by determining the extent of missing data and use logistic regression to identify factors associated with missing data. We will conduct sensitivity analysis using pattern-mixture and selection models under missing not at random (MNAR) assumptions to examine the robustness of conclusions of the primary analysis to missing data.

SECTION VI: RESEARCH INVOLVING DRUGS, BIOLOGICS, RADIOTRACERS, PLACEBOS AND DEVICES

If this section (or one of its parts, A or B) is not applicable, state N/A and delete the rest of the section.
N/A

SECTION VII: RECRUITMENT/CONSENT AND ASSENT PROCEDURES

1. **Targeted Enrollment: Give the number of subjects:**
   a. targeted for enrollment at Yale for this protocol_25
   b. If this is a multi-site study, give the total number of subjects targeted across all sites_270__

2. **Indicate recruitment methods below.** Attach copies of any recruitment materials that will be used.

- ☐ Flyers
- ☐ Posters
- ☐ Letter
- ☐ Medical Record Review
- ☐ Departmental/Center Newsletters
- ☐ YCCI Recruitment database
- ☐ Other (describe):
- ☐ Internet/Web Postings
- ☐ Mass E-mail Solicitation
- ☐ Departmental/Center Website
- ☐ Departmental/Center Research Boards
- ☐ Web-Based Clinical Trial Registries
- ☐ Clinicaltrials.gov Registry (do not send materials to HIC)
- ☐ Radio
- ☐ Telephone
- ☐ Television
- ☐ Newspaper

3. **Recruitment Procedures:**
   a. Describe how potential subjects will be identified.

I. Focus Groups:
Local Research Assistants will work with the Site PIs and providers to identify patients, staff and payers for the focus groups from their HIV clinical care sites.
II. Online Survey:
Local Research Assistants will work with the Site PIs to identify clinic leadership staff and providers.
III. Patient Outcomes:

To assess the impact of the Implementation Facilitation, patients will not be directly recruited. We will use electronic medical records at each site to evaluate the effectiveness of the implementation facilitation strategy on patient level outcomes.

b. Describe how potential subjects are contacted.

I. Focus Groups:
Local Research Assistants, Site PIs and providers will approach potential subjects for participation in focus groups.

II. Online Survey:
Yale research staff and local site PIs will contact clinic leadership staff and providers for surveys using emails.
III. Patient Outcomes:
Patients will not be directly recruited as we will rely on data available through the electronic medical record collected as part of routine care.

c. Who is recruiting potential subjects?

I. Focus Groups:
Local Research Assistants, Site PIs and providers will recruit potential subjects for participation in focus groups.
II. Online Survey:
Yale research staff and local site PIs will recruit clinic leadership staff and providers for surveys using emails.
III. Patient Outcomes:
N/a

4. Screening Procedures

a. Will email or telephone correspondence be used to screen potential subjects for eligibility prior to the potential subject coming to the research office? ☐ Yes ☒ No
b. If yes, identify below all health information to be collected as part of screening and check off any of the following HIPAA identifiers to be collected and retained by the research team during this screening process.

HEALTH INFORMATION TO BE COLLECTED:
HIPAA identifiers:
☐ Names
☐ All geographic subdivisions smaller than a State, including: street address, city, county, precinct, zip codes and their equivalent geocodes, except for the initial three digits of a zip code if, according to the current publicly-available data from the Bureau of the Census: (1) the geographic unit formed by combining all zip codes with the same three initial digits contains more than 20,000 people, and (2) the initial three digits of a zip code for all such geographic units containing 20,000 or fewer people is changed to 000.
☐ Telephone numbers
☐ Fax numbers
☐ E-mail addresses
☐ Social Security numbers
☐ Medical record numbers
☐ Health plan beneficiary numbers

☐ Account numbers
☐ All elements of dates (except year) for dates related to an individual, including: birth date, admission date, discharge date, date of death, all ages over 89 and all elements of dates (including year) indicative of such age, except that such ages and elements may be aggregated into a single category of age 90 or older
☐ Certificate/license numbers
☐ Vehicle identifiers and serial numbers, including license plate numbers
☐ Device identifiers and serial numbers
☐ Web Universal Resource Locators (URLs)
☐ Internet Protocol (IP) address numbers
☐ Biometric identifiers, including finger and voice prints
☐ Full face photographic images and any comparable images
☐ Any other unique identifying numbers, characteristics, or codes

5. **Assessment of Current Health Provider Relationship for HIPAA Consideration:**
Does the Investigator or any member of the research team have a direct existing clinical relationship with any potential subject?
☐ Yes, all subjects
☒ Yes, some of the subjects
☐ No

If yes, describe the nature of this relationship.

The Yale investigators may have a clinical relationship with subject potential subjects who participate in the piloting of interview guides for the focus groups. The local Site PI's may have a clinical relationship with a potential subject.

6. **Request for waiver of HIPAA authorization:** (When requesting a waiver of HIPAA Authorization for either the entire study, or for recruitment purposes only. Note: if you are collecting PHI as part of a phone or email screen, you must request a HIPAA waiver for recruitment purposes.)

Choose one:
☒ For entire study (Patient outcomes component only, see below)
☐ For recruitment purposes only
☐ For inclusion of non-English speaking subject if short form is being used

i. Describe why it would be impracticable to obtain the subject's authorization for use/disclosure of this data;
ii. If requesting a waiver of **signed** authorization, describe why it would be impracticable to obtain the subject's signed authorization for use/disclosure of this data;

I. Focus Groups: No personally identifying information will be collected; therefore, no HIPPA form is required.

II. Online Survey: No personally identifying information will be collected therefore, no HIPPA form is required.

III. Patient Outcomes: Electronic medical record data will be collected in aggregate and de-identified when possible. A HIPPA waiver is requested.

By signing this protocol application, the investigator assures that the protected health information for which a Waiver of Authorization has been requested will not be reused or disclosed to any person or entity other than those listed in this application, except as required by law, for authorized oversight of this research study, or as specifically approved for use in another study by an IRB.

Researchers are reminded that unauthorized disclosures of PHI to individuals outside of the Yale HIPAA-Covered entity must be accounted for in the "accounting for disclosures log", by subject name, purpose, date, recipients, and a description of information provided. Logs are to be forwarded to the Deputy HIPAA Privacy Officer.

7. **Required HIPAA Authorization:** If the research involves the creation, use or disclosure of protected health information (PHI), separate subject authorization is required under the HIPAA Privacy Rule. Indicate which of the following forms are being provided:
   - ☐ Compound Consent and Authorization form
   - ☐ HIPAA Research Authorization Form

8. **Consent Personnel:** List the names of all members of the research team who will be obtaining consent/assent.

David Fiellin, MD
Lynn Fiellin, MD
E. Jennifer Edelman, MD
Jeanette Tetrault, MD
Philip Chan, MD
Deborah Cornman, PhD
Gabriel Rebick, MD
Evangelia Louizos
Michael Virata, MD
Research Assistants to be hired

9. **Process of Consent/Assent:** Describe the setting and conditions under which consent/assent will be obtained, including parental permission or surrogate permission and the steps taken to ensure subjects' independent decision-making.

I. Focus Groups:
The Site PI or Research Assistant will obtain informed consent from patients and site staff participating in the focus group interviews. Participants will be asked to come to a private office where they will meet with a Research Assistant who will describe the study in more detail, determine interest in participating, and assess potential eligibility. After fully informing participants about the study and answering any questions, the Research Assistant will obtain verbal consent.

II. Online Survey:
Yale research staff and local site PIs will contact clinic leadership staff and providers for surveys using emails and participants will consent to the study online before taking the survey.

III. Patient Outcomes:
Patients will not be directly approached; a waiver is requested.

10. E**valuation of Subject(s) Capacity to Provide Informed Consent/Assent:** Indicate how the personnel obtaining consent will assess the potential subject's ability and capacity to consent to the research being proposed.

    All research personnel are required to participate in and document training in protection of human subjects and the responsible conduct of scientific research, including assessment of a potential subject's capacity to consent to the study.

11. **Documentation of Consent/Assent:** Specify the documents that will be used during the consent/assent process. Copies of all documents should be appended to the protocol, in the same format that they will be given to subjects.

    I. Focus Groups:
    A verbal informed consent will be used for focus groups.
    II. Online Survey:
    An online informed consent will be used for survey data.
    III. Patient Outcomes:
    Electronic Medical Records will be used; therefore, is a waiver of informed consent is requested.

12. **Non-English Speaking Subjects:** Explain provisions in place to ensure comprehension for research involving non-English speaking subjects. If enrollment of these subjects is anticipated, translated copies of all consent materials must be submitted for approval prior to use.

    N/A

    12(a) As a limited alternative to the above requirement, will you use the short form* for consenting process if you unexpectedly encounter a non-English speaking individual interested in study participation and the translation of the long form is not possible prior to intended enrollment?
    YES ☐ NO ☐

    Note* If more than 2 study participants are enrolled using a short form translated into the same language, then the full consent form should be translated into that language for use the next time a subject speaking that language is to be enrolled.

    Several translated short form templates are found on our website at: http://www.yale.edu/hrpp/forms-templates/biomedical.html. If the translation of the short

form is not available on our website, then the translated short form needs to be submitted to the IRB office for approval via amendment prior to enrolling the subject. ***Please review the guidance and presentation on use of the short form available on the HRPP website.***

If using a short form without a translated HIPAA Research Authorization Form, please request a HIPAA waiver in the section above.

(Note to HIC: This section is repeated for each of the 3 aspects of the study, 13a, 13b, 13c)

13a. Consent Waiver: In certain circumstances, the HIC may grant a waiver of signed consent, or a full waiver of consent, depending on the study. If you will request either a waiver of consent, or a waiver of signed consent for this study, complete the appropriate section below.

I. Focus Groups:
Requesting a waiver of signed consent; a verbal informed consent will be used.

☐ **Not Requesting a consent waiver**
☒ **Requesting a waiver of signed consent**
☐ **Requesting a full waiver of consent**

A. Waiver of **signed** consent: (Verbal consent from subjects will be obtained. **If PHI is collected, information in this section must match Section VII, Question 6**)
☒ **Requesting a waiver of signed consent for Recruitment/Screening only**

If requesting a waiver of signed consent, please address the following:
a. Would the signed consent form be the only record linking the subject and the research?
☒ Yes ☐ No
b. Does a breach of confidentiality constitute the principal risk to subjects?
☒ Yes ☐ No

OR

c. Does the research activity pose greater than minimal risk?
☐ Yes ***If you answered yes, stop. A waiver cannot be granted.*** Please note: Recruitment/screening is generally a minimal risk research activity
☐ No

AND

d. Does the research include any activities that would require signed consent in a non-research context? ☐ Yes ☒ No

☐ **Requesting a waiver of signed consent for the Entire Study (**Note that an information sheet may be required.)

If requesting a waiver of signed consent, please address the following:
a. Would the signed consent form be the only record linking the subject and the research?
☐ Yes ☐ No
b. Does a breach of confidentiality constitute the principal risk to subjects?
☐ Yes ☐ No

OR

c. Does the research pose greater than minimal risk? ☐ Yes ***If you answered yes, stop. A waiver cannot be granted.*** ☐ No

AND

d. Does the research include any activities that would require signed consent in a non-research context? ☐ Yes ☐ No

B. **Full waiver** of consent: (No consent from subjects will be obtained for the activity.)

☐ **Requesting a waiver of consent for Recruitment/Screening only**

a. Does the research activity pose greater than minimal risk to subjects?
☐ Yes ***If you answered yes, stop. A waiver cannot be granted.*** Please note: Recruitment/screening is generally a minimal risk research activity
☐ No
b. Will the waiver adversely affect subjects' rights and welfare? ☐ Yes ☐ No
c. Why would the research be impracticable to conduct without the waiver?
d. Where appropriate, how will pertinent information be returned to, or shared with subjects at a later date?

☐ **Requesting a full waiver of consent for the Entire Study (Note: If PHI is collected, information here must match Section VII, question 6.)**

If requesting a full waiver of consent, please address the following:

a. Does the research pose greater than minimal risk to subjects?
☐ Yes ***If you answered yes, stop. A waiver cannot be granted.***
☐ No
b. Will the waiver adversely affect subjects' rights and welfare? ☐ Yes ☐ No
c. Why would the research be impracticable to conduct without the waiver?
d. Where appropriate, how will pertinent information be returned to, or shared with subjects at a later date

13b. Consent Waiver: In certain circumstances, the HIC may grant a waiver of signed consent, or a full waiver of consent, depending on the study. If you will request either a waiver of consent, or a waiver of signed consent for this study, complete the appropriate section below.

☐ **Not Requesting a consent waiver**
☒ **Requesting a waiver of signed consent**
☐ **Requesting a full waiver of consent**

II. Online Survey:
Requesting a waiver of signed consent; an online informed consent will be used.

A. Waiver of **signed** consent: (Verbal consent from subjects will be obtained. **If PHI is collected, information in this section must match Section VII, Question 6**)

☒ **Requesting a waiver of signed consent for Recruitment/Screening only**

If requesting a waiver of signed consent, please address the following:

a. Would the signed consent form be the only record linking the subject and the research?
☒ Yes ☐ No

b. Does a breach of confidentiality constitute the principal risk to subjects?
☒ Yes ☐ No

OR

c. Does the research activity pose greater than minimal risk?
☐ Yes ***If you answered yes, stop. A waiver cannot be granted.*** Please note: Recruitment/screening is generally a minimal risk research activity
☐ No

AND

d. Does the research include any activities that would require signed consent in a non-research context? ☐ Yes ☒ No

☐ **Requesting a waiver of signed consent for the Entire Study (**Note that an information sheet may be required.)

If requesting a waiver of signed consent, please address the following:

a. Would the signed consent form be the only record linking the subject and the research?
☐ Yes ☐ No

b. Does a breach of confidentiality constitute the principal risk to subjects?
☐ Yes ☐ No

OR

c. Does the research pose greater than minimal risk? ☐ Yes ***If you answered yes, stop. A waiver cannot be granted.*** ☐ No

AND

d. Does the research include any activities that would require signed consent in a non-research context? ☐ Yes ☐ No

B. **Full waiver** of consent: (No consent from subjects will be obtained for the activity.)

☐ **Requesting a waiver of consent for Recruitment/Screening only**

a. Does the research activity pose greater than minimal risk to subjects?
☐ Yes ***If you answered yes, stop. A waiver cannot be granted.*** Please note: Recruitment/screening is generally a minimal risk research activity
☐ No

b. Will the waiver adversely affect subjects' rights and welfare? ☐ Yes ☐ No

c. Why would the research be impracticable to conduct without the waiver?

d. Where appropriate, how will pertinent information be returned to, or shared with subjects at a later date?

☐ **Requesting a full waiver of consent for the Entire Study (Note: If PHI is collected, information here must match Section VII, question 6.)**

If requesting a full waiver of consent, please address the following:

a. Does the research pose greater than minimal risk to subjects?
☐ Yes ***If you answered yes, stop. A waiver cannot be granted.***
☒ No
b. Will the waiver adversely affect subjects' rights and welfare? ☐ Yes ☐ No
c. Why would the research be impracticable to conduct without the waiver?
d. Where appropriate, how will pertinent information be returned to, or shared with subjects at a later date?

13c. Consent Waiver: In certain circumstances, the HIC may grant a waiver of signed consent, or a full waiver of consent, depending on the study. If you will request either a waiver of consent, or a waiver of signed consent for this study, complete the appropriate section below.

III. Patient Outcomes:
Electronic Medical Records will be used; there is a waiver of consent.

☐ **Not Requesting a consent waiver**
☐ **Requesting a waiver of signed consent**
☒ **Requesting a full waiver of consent**

A. Waiver of **signed** consent: (Verbal consent from subjects will be obtained. **If PHI is collected, information in this section must match Section VII, Question 6**)
☐ **Requesting a waiver of signed consent for Recruitment/Screening only**
If requesting a waiver of signed consent, please address the following:
a. Would the signed consent form be the only record linking the subject and the research?
☐ Yes ☐ No
b. Does a breach of confidentiality constitute the principal risk to subjects?
☐ Yes ☐ No

OR

c. Does the research activity pose greater than minimal risk?
☐ Yes ***If you answered yes, stop. A waiver cannot be granted.*** Please note: Recruitment/screening is generally a minimal risk research activity
☐ No

AND

d. Does the research include any activities that would require signed consent in a non-research context? ☐ Yes ☐ No

☐ **Requesting a waiver of signed consent for the Entire Study (**Note that an information sheet may be required.)

If requesting a waiver of signed consent, please address the following:
a. Would the signed consent form be the only record linking the subject and the research?
☐ Yes ☐ No
b. Does a breach of confidentiality constitute the principal risk to subjects?
☐ Yes ☐ No

OR

c. Does the research pose greater than minimal risk? ☐ Yes ***If you answered yes, stop. A waiver cannot be granted.*** ☐ No

AND

d. Does the research include any activities that would require signed consent in a non-research context? ☐ Yes ☐ No

B. **Full waiver** of consent: (No consent from subjects will be obtained for the activity.)
☐ **Requesting a waiver of consent for Recruitment/Screening only**
a. Does the research activity pose greater than minimal risk to subjects?
☐ Yes ***If you answered yes, stop. A waiver cannot be granted.*** Please note: Recruitment/screening is generally a minimal risk research activity
☐ No
b. Will the waiver adversely affect subjects' rights and welfare? ☐ Yes ☐ No
c. Why would the research be impracticable to conduct without the waiver?
d. Where appropriate, how will pertinent information be returned to, or shared with subjects at a later date?

☒ **Requesting a full waiver of consent for the Entire Study (Note: If PHI is collected, information here must match Section VII, question 6.)**

If requesting a full waiver of consent, please address the following:

a. Does the research pose greater than minimal risk to subjects?
☐ Yes ***If you answered yes, stop. A waiver cannot be granted.***
☒ No
b. Will the waiver adversely affect subjects' rights and welfare? ☐ Yes ☒ No
c. Why would the research be impracticable to conduct without the waiver? It is not feasible to obtain consent from all participants.
d. Where appropriate, how will pertinent information be returned to, or shared with subjects at a later date? N/A

SECTION VIII: PROTECTION OF RESEARCH SUBJECTS

Confidentiality & Security of Data:

a. What protected health information (medical information along with the HIPAA identifiers) about subjects will be collected and used for the research?

Electronic medical record data: We will only collect aggregate and de-identified data from the electronic medical record. Any potentially individual identifiable health information will be protected in accordance with the Health Insurance Portability and Accountability Act (HIPAA) of 1996. All research personnel will be trained on Institutional Review Board (IRB) and HIPAA procedures.

b. How will the research data be collected, recorded and stored?

Recorded sessions: Participants will be made aware during the informed consent process that focus groups will be digitally recorded. However, participants will be instructed to avoid using names and the recordings will be coded by randomly generated group numbers to protect participant confidentiality. Participants will be given the option to have their information redacted from transcripts. The transcripts will be reviewed by Dr. Edelman or the Research Coordinator and all personally identifying information will be removed prior to analysis. All appropriate actions will be taken by staff members to minimize the risks associated with loss of confidentiality. Audio files will be coded by number and will be erased. Audio recordings will be stored on a password protected computer. The research team and the professional transcriptionists will use the Yale Secure Transfer File website to send recordings and transcripts.

Survey information: Information obtained from sites will include descriptive information and research assessments. Access to this information will be limited to study staff. Analytic datasets will be created with study identification codes.

Electronic medical record data: We will only collect aggregate and de-identified data from the electronic medical record. Any potentially individual identifiable health information will be protected in accordance with the Health Insurance Portability and Accountability Act (HIPAA) of 1996.

c. How will the digital data be stored? ☐ CD ☐ DVD ☐ Flash Drive ☐ Portable Hard Drive ☒ Secured Server ☐ Laptop Computer ☐ Desktop Computer ☐ Other

d. What methods and procedures will be used to safeguard the confidentiality and security of the identifiable study data and the storage media indicated above during and after the subject's participation in the study?

We are planning to use a Web-based computer system for data collection, monitoring and reporting. This system has proven to be very efficient and reliable in clinical trials being conducted by our research team. The core of this system consists of a database hosted on a network of secure servers that are capable of collecting and storing data using Web based applications that are accessed by the remote users via standard Internet browsers. The Web

based data system is designed to fulfill strict requirements of a clinical trial regarding data collection, monitoring, and reporting from the recruitment of participants to the delivery of data sets suitable for statistical analyses at the end of the trial. The system meets the highest security and reliability standards. All connections to the systems are secured and encrypted and only authorized users are able to access the system. All data is stored in encrypted files, and multiple backups of the system and all data are maintained.
Identifying information from any of the participants in the formative evaluations will be kept separately from any forms on which they record information. References to identifying information will be eliminated from the written transcript of the interview in preparation for analysis of qualitative data. Names and any other identifying information collected on evaluation forms, meeting minutes, or field notes and document reviews will be eliminated in preparation for analysis of these records. These precautions are expected to be completely effective in eliminating risks to confidentiality.

Information obtained from sites will include descriptive information and research assessments. Access to this information will be limited to study staff. Analytic datasets will be created with study identification codes.

Do all portable devices contain encryption software? ☒ Yes ☐ No
If no, see http://hipaa.yale.edu/guidance/policy.html

e. What will be done with the data when the research is completed? Are there plans to destroy the identifiable data? If yes, describe how, by whom and when identifiers will be destroyed. If no, describe how the data and/or identifiers will be secured.

Upon completion of the study, all computerized subject datasets will be stored in a password-protected study computer, to which only the PI, investigators and study personnel will have access. All paper files with subject information will remain in locked files in the study office of the PI, until they are destroyed, after all analyses are complete.

f. Who will have access to the protected health information (such as the research sponsor, the investigator, the research staff, all research monitors, FDA, Yale Cancer Center Data and Safety Monitoring Committee (DSMC), SSC, etc.)? (please distinguish between PHI and de-identified data)

During an audit or program evaluation, representatives from the Yale Human Investigation Committee and from the National Institutes of Health may have access to subject data, but will strictly follow rules of confidentiality.

g. If appropriate, has a Certificate of Confidentiality been obtained?

We have obtained a Certificate of Confidentiality from the National Institute on Drug Abuse.

h. Are any of the study procedures likely to yield information subject to mandatory reporting requirements? (e.g. HIV testing – reporting of communicable diseases; parent interview -incidents of child abuse, elderly abuse, etc.). Please verify to whom such instances will need to be reported.

No.

SECTION IX: POTENTIAL BENEFITS

Potential Benefits: Identify any benefits that may be reasonably expected to result from the research, either to the subject(s) or to society at large. (Payment of subjects is not considered a benefit in this context of the risk benefit assessment.)

Participants and the HIV clinics will gain from their feedback from the academic detailing, provider education, stakeholder engagement, program tailoring, performance monitoring and feedback, formative evaluation, learning collaborative, and program marketing. Participants and others may eventually benefit from the scientific knowledge that may be gained from this work.

SECTION X: RESEARCH ALTERNATIVES AND ECONOMIC CONSIDERATIONS

1. **Alternatives:** What other alternatives are available to the study subjects outside of the research?

   Potential study subjects have the freedom to refuse participation in the focus groups.
2. **Payments for Participation (Economic Considerations):** Describe any payments that will be made to subjects, the amount and schedule of payments, and the conditions for receiving this compensation.

   Patients will be compensated $25 for their participation in focus groups. As allowed by local institutional policies, clinic providers, administrators and payers will be compensated $50 for their participation.

3. **Costs for Participation (Economic Considerations):** Clearly describe the subject's costs associated with participation in the research, and the interventions or procedures of the study that will be provided at no cost to subjects.

   There are no costs to the subjects to participate in this research.

4. **In Case of Injury:** This section is required for any research involving more than minimal risk.
   N/A
   a. Will medical treatment be available if research-related injury occurs?
   b. Where and from whom may treatment be obtained?
   c. Are there any limits to the treatment being provided?
   d. Who will pay for this treatment?
   e. How will the medical treatment be accessed by subjects?
