# Supplementary material for: Effect of Implementation Facilitation to Promote Adoption of Medications for Addiction Treatment in US HIV Clinics: A Randomized Clinical Trial
Source: JAMA Netw Open. 2022 Oct 17;5(10):e2236904. doi: 10.1001/jamanetworkopen.2022.36904 (PMC9577676; doi:10.1001/jamanetworkopen.2022.36904)
Supplement: Supplement 2. — eFigure 1. CONSORT Flow Diagram eTable 1. Medications Available for HIV Clinic-Based Addiction Treatment by Substance Use Disorder eTable 2. Clinician and Staff Readiness to Provide MAT by Study Period, Results From GEE eTable 3. Clinician and Staff ORCA Evidence Ratings for MAT by Study Period, Results From GEE eFigure 2. Clinician and Staff ORCA Evidence Ratings for Medications for Addiction Treatment by Study Period eTable 4. Clinician and Staff ORCA Context Ratings for MAT by Study Period, Results From GEE eFigure 3. Clinician and Staff ORCA Context Ratings for Medications for Addiction Treatment by Study Period [file jamanetwopen-e2236904-s002.pdf]

Supplemental Online Content

Edelman EJ, Gan G, Dziura J, et al. Effect of implementation facilitation to promote adoption of medications for addiction treatment in US HIV clinics: a randomized clinical trial. *JAMA Netw Open*. 2022;5(10):e2236904. doi:10.1001/jamanetworkopen.2022.36904

eFigure 1. CONSORT Flow Diagram
eTable 1. Medications Available for HIV Clinic-Based Addiction Treatment by Substance Use Disorder
eTable 2. Clinician and Staff Readiness to Provide MAT by Study Period, Results From GEE
eTable 3. Clinician and Staff ORCA Evidence Ratings for MAT by Study Period, Results From GEE
eFigure 2. Clinician and Staff ORCA Evidence Ratings for Medications for Addiction Treatment by Study Period
eTable 4. Clinician and Staff ORCA Context Ratings for MAT by Study Period, Results From GEE
eFigure 3. Clinician and Staff ORCA Context Ratings for Medications for Addiction Treatment by Study Period

This supplemental material has been provided by the authors to give readers additional information about their work.

© 2022 Edelman EJ et al. *JAMA Network Open*.

eFigure 1. CONSORT Flow Diagram

CONSORT
TRANSPARENT REPORTING of TRIALS

CONSORT 2010 Flow Diagram

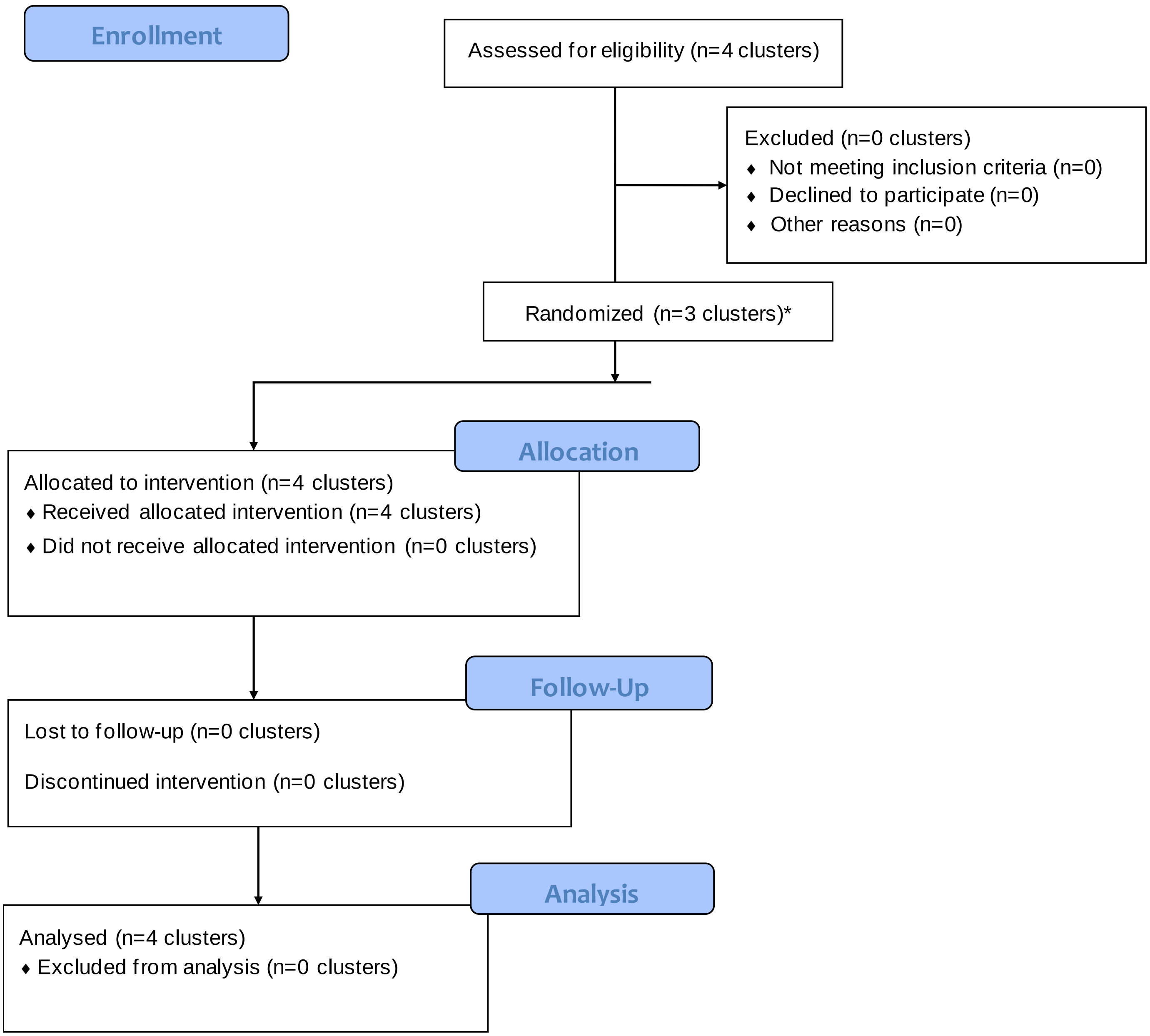

*One cluster not randomized to order of intervention delivery.

© 2022 Edelman EJ et al. *JAMA Network Open*.

eTable 1. Medications Available for HIV Clinic-Based Addiction Treatment by Substance Use Disorder*

Substance Use Disorder	Medications for Addiction Treatment
Opioid Use Disorder	• Buprenorphine products<br>• Naltrexone, injectable formulation
Alcohol Use Disorder	• Disulfiram<br>• Acamprosate<br>• Naltrexone, injectable and oral formulations
Tobacco Use Disorder	• Nicotine replacement therapy, all products<br>• Varenicline<br>• Bupropion

Asterisk (*): Reflects medications included in WHAT-IF? primary outcome

© 2022 Edelman EJ et al. *JAMA Network Open.*

eTable 2. Clinician and Staff Readiness to Provide MAT by Study Period, Results From GEE

Study period	Readiness to provide MOUD			Readiness to provide MAUD			Readiness to provide MTUD		
	N	Overall	p value	N	Overall	p value	N	Overall	p value
Control	69	6·79	Ref	69	6·47	ref	69	7·59	ref
Intervention	59	6·59	0·62	59	6·16	0·47	59	7·16	0·19
Evaluation	58	7·19	0·32	58	7·09	0·13	58	7·52	0·86
Maintenance	88	6.96	0·66	88	6·65	0·68	88	7·45	0·69

Note: MOUD=medications for opioid use disorder, MAUD=medications for alcohol use disorder, MTUD=medications for tobacco use disorder

© 2022 Edelman EJ et al. *JAMA Network Open*.

eTable 3. Clinician and Staff ORCA Evidence Ratings for MAT by Study Period, Results From GEE

Study period	Evidence rating for MOUD		Evidence rating for MAUD		Evidence rating for MTUD	
	**Overall**	**p value**	**Overall**	**p value**	**Overall**	**p value**
Control	4·36	Ref	3·90	ref	4·22	ref
Intervention	4·40	0·76	4·11	0·09	4·25	0·83
Evaluation	4·37	0·94	4·07	0·23	4·10	0·44
Maintenance	4·34	0·87	4·19	0·01	4·30	0·48

Note: ORCA=organizational readiness to change assessment, MOUD=medications for opioid use disorder, MAUD=medications for alcohol use disorder, MTUD=medications for tobacco use disorder

© 2022 Edelman EJ et al. *JAMA Network Open.*

eFigure 2. Clinician and Staff ORCA Evidence Ratings for Medications for Addiction Treatment by Study Period*

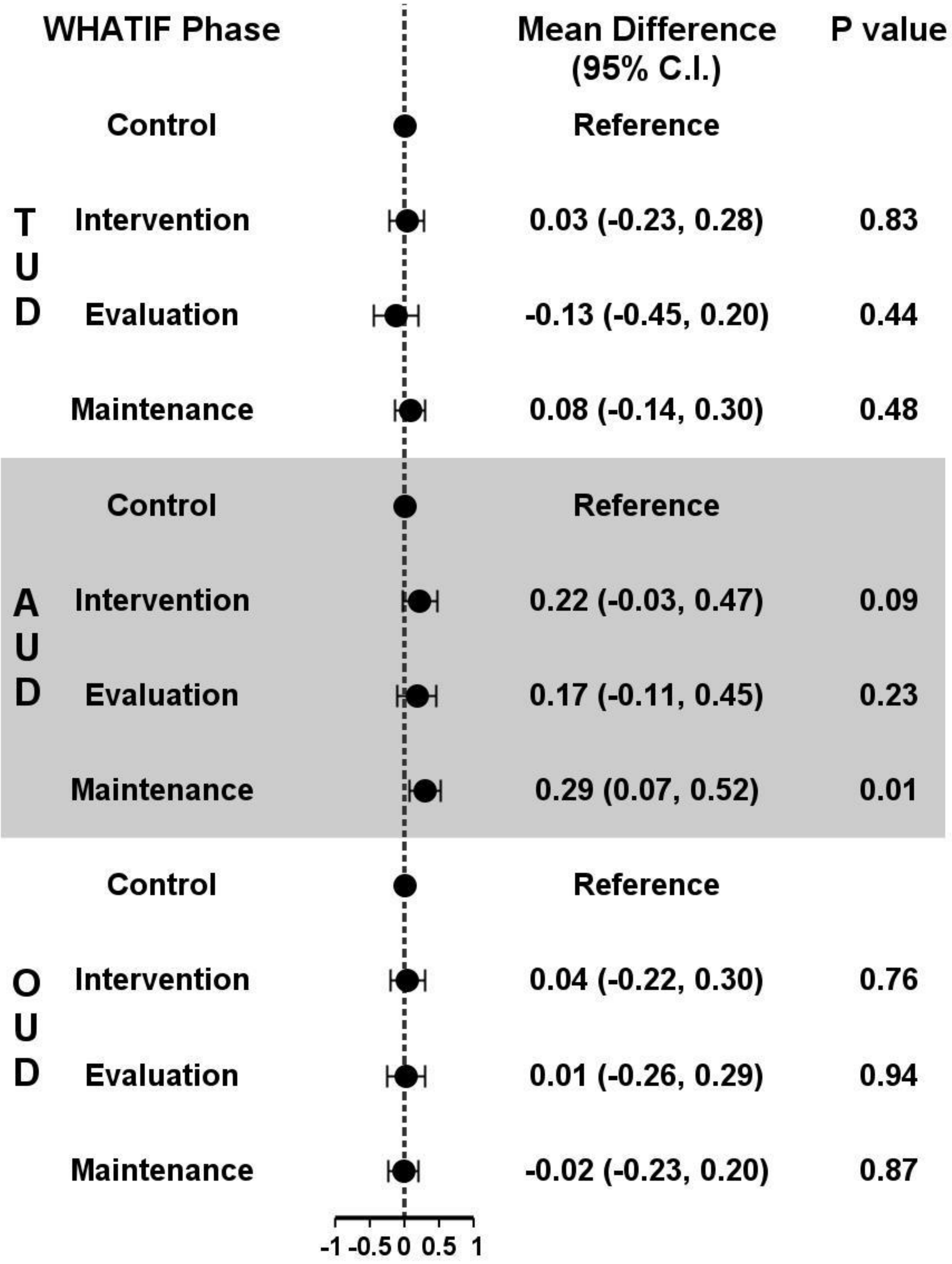

*Results from generalized estimating equation (GEE) models. ORCA=organizational readiness to change assessment.

© 2022 Edelman EJ et al. *JAMA Network Open.*

eTable 4. Clinician and Staff ORCA Context Ratings for MAT by Study Period, Results From GEE

Study period	Leadership Culture		Staff Culture		Leadership Practice		Evaluation Accountability		Opinion Leader Culture		Slack Resources	
	**Overall**	**p value**	**Overall**	**p value**	**Overall**	**p value**	**Overall**	**p value**	**Overall**	**p value**	**Overall**	**p value**
Control	3·72	ref	4·08	ref	3·90	ref	3·81	Ref	3·81	ref	3·30	ref
Intervention	3·76	0·70	4·19	0·26	3·81	0·41	3·94	0·24	3·95	0·21	3·33	0·74
Evaluation	3·65	0·60	4.04	0·73	3·80	0·41	3·81	0·96	3·88	0·63	3·33	0·78
Maintenance	3·67	0·66	4.07	0·89	3·79	0·35	3·72	0·42	3·85	0·74	3·40	0·35

Note: ORCA=organizational readiness to change assessment, MOUD=medications for opioid use disorder, MAUD=medications for alcohol use disorder, MTUD=medications for tobacco use disorder

© 2022 Edelman EJ et al. *JAMA Network Open.*

eFigure 3. Clinician and Staff ORCA Context Ratings for Medications for Addiction Treatment by Study Period*

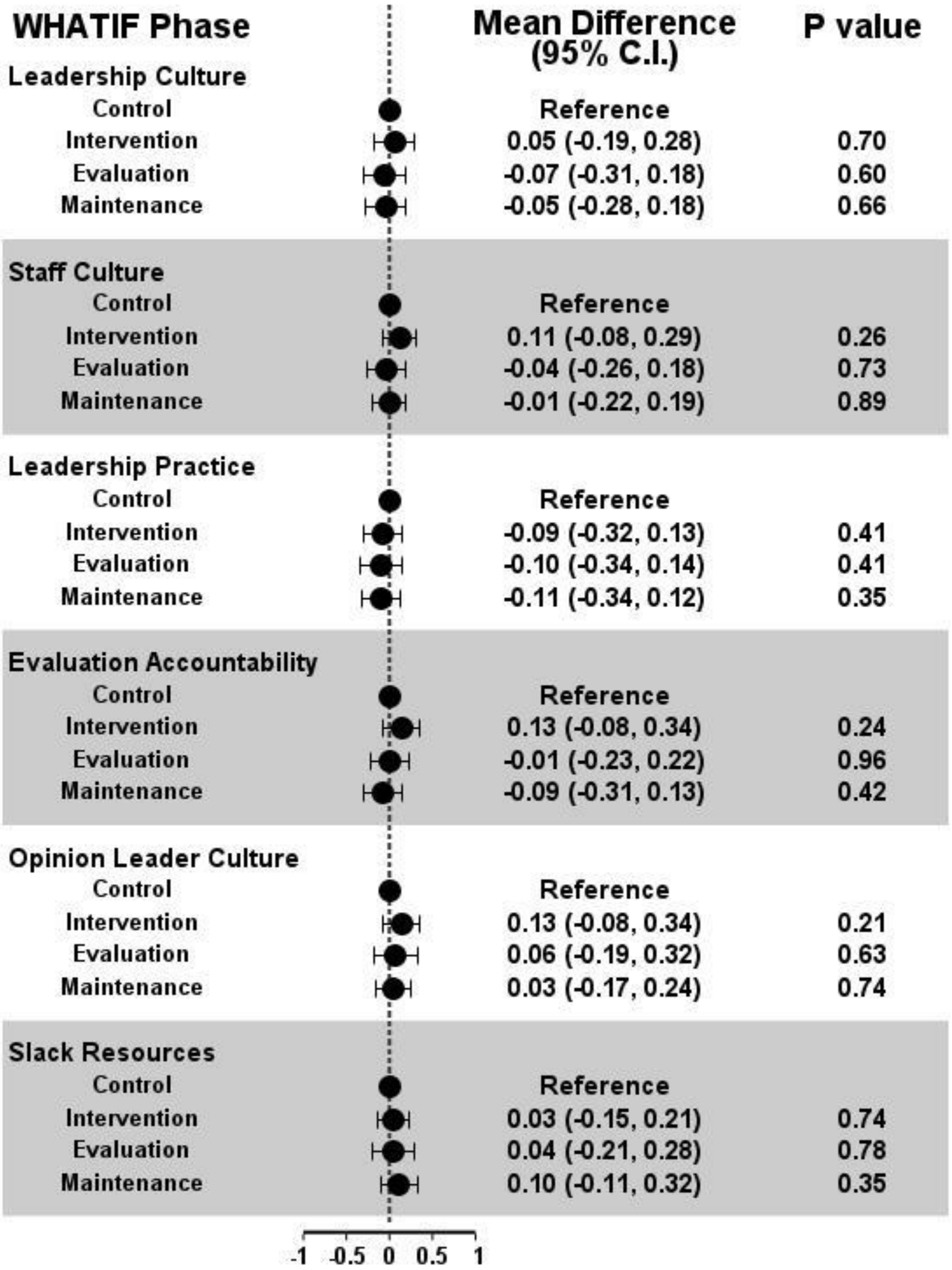

*Results from generalized estimating equation (GEE) models. ORCA=organizational readiness to change assessment.

© 2022 Edelman EJ et al. *JAMA Network Open*.
