# Supplementary material for: Effect of Implementation Facilitation to Promote Adoption of Medications for Addiction Treatment in US HIV Clinics: A Randomized Clinical Trial
Source: JAMA Netw Open. 2022 Oct 17;5(10):e2236904. doi: 10.1001/jamanetworkopen.2022.36904 (PMC9577676; doi:10.1001/jamanetworkopen.2022.36904)
Supplement: Supplement 3. — Data Sharing Statement [file jamanetwopen-e2236904-s003.pdf]

Data Sharing Statement

Edelman. Effect of Implementation Facilitation to Promote Adoption of Medications for Addiction Treatment in US HIV Clinics. *JAMA Netw Open*. Published October 17, 2022. doi:10.1001/jamanetworkopen.2022.36904

Data
Data available: Yes
Data types: Other (please specify)
Additional Information: Deidentified data and data dictionary will be made available upon approval by the PI and study team.
How to access data: upon written request
When available: With publication

Supporting Documents
Document types: Other (please specify)
Additional Information: Protocol paper has been published at Contemporary Clinical Trials and includes protocol details alon with analytic plan and data collection tools as supplementary material; happy to make other documents available upon request.
How to access documents: https://www.sciencedirect.com/science/article/pii/S1551714420302342?via%3Dihub
When available: With publication

Additional Information
Who can access the data: upon approval by PI
Types of analyses: when relevant for proposed reserach question
Mechanisms of data availability: after approval and with a DUA as indicated
